# Direct C–H trifluoromethylation of di- and trisubstituted alkenes by photoredox catalysis

**DOI:** 10.3762/bjoc.10.108

**Published:** 2014-05-12

**Authors:** Ren Tomita, Yusuke Yasu, Takashi Koike, Munetaka Akita

**Affiliations:** 1 Chemical Resources Laboratory, Tokyo Institute of Technology, R1-27, 4259 Nagatsuta, Midori-ku, Yokohama 226-8503, Japan

**Keywords:** electrophilic trifluoromethylating reagent, multi-substituted alkene, photoredox catalysis, radical reaction, trifluoromethylation

## Abstract

**Background:** Trifluoromethylated alkene scaffolds are known as useful structural motifs in pharmaceuticals and agrochemicals as well as functional organic materials. But reported synthetic methods usually require multiple synthetic steps and/or exhibit limitation with respect to access to tri- and tetrasubstituted CF_3_-alkenes. Thus development of new methodologies for facile construction of C_alkenyl_–CF_3_ bonds is highly demanded.

**Results:** The photoredox reaction of alkenes with 5-(trifluoromethyl)dibenzo[*b*,*d*]thiophenium tetrafluoroborate, Umemoto’s reagent, as a CF_3_ source in the presence of [Ru(bpy)_3_]^2+^ catalyst (bpy = 2,2’-bipyridine) under visible light irradiation without any additive afforded CF_3_-substituted alkenes via direct C_alkenyl_–H trifluoromethylation. 1,1-Di- and trisubstituted alkenes were applicable to this photocatalytic system, providing the corresponding multisubstituted CF_3_-alkenes. In addition, use of an excess amount of the CF_3_ source induced double C–H trifluoromethylation to afford geminal bis(trifluoromethyl)alkenes.

**Conclusion:** A range of multisubstituted CF_3_-alkenes are easily accessible by photoredox-catalyzed direct C–H trifluoromethylation of alkenes under mild reaction conditions. In particular, trifluoromethylation of triphenylethene derivatives, from which synthetically valuable tetrasubstituted CF_3_-alkenes are obtained, have never been reported so far. Remarkably, the present facile and straightforward protocol is extended to double trifluoromethylation of alkenes.

## Introduction

The trifluoromethyl (CF_3_) group is a useful structural motif in many bioactive molecules as well as functional organic materials [1–6]. Thus, the development of new methodologies for highly efficient and selective incorporation of a CF_3_ group into diverse skeletons has become a hot research topic in the field of organic synthetic chemistry [7–12]. Recently, radical trifluoromethylation by photoredox catalysis [13–23] with ruthenium(II) polypyridine complexes (e.g., [Ru(bpy)_3_]^2+^ (bpy: 2,2’-bipyridine)), the relevant Ir cyclometalated complexes (e.g., *fac*-Ir(ppy)_3_ (ppy: 2-phenylpyridine)) and organic dyes has been developed; the trifluoromethyl radical (·CF_3_) can be easily generated from conventional CF_3_ radical precursors such as CF_3_I, CF_3_SO_2_Cl and CF_3_SO_2_Na through visible-light-induced single-electron transfer (SET) processes [24–32]. On the other hand, we have intensively developed trifluoromethylations of olefins by the Ru and Ir photoredox catalysis using easy-handling and shelf-stable electrophilic trifluoromethylating reagents [33–36] (***^+^CF_3_***) such as Umemoto’s reagent (**1a**, 5-(trifluoromethyl)dibenzo[*b*,*d*]thiophenium tetrafluoroborate) and Togni’s reagents **1b** (1-(trifluoromethyl)-1λ^3^,2-benziodoxol-3(1*H*)-one) and **1c** (3,3-dimethyl-1,3-dihydro-1λ^3^,2-benziodoxole) [37–41]. It was found that electrophilic trifluoromethylating reagents (***^+^CF_3_***) can serve as more efficient CF_3_ radical sources under mild photocatalytic reaction conditions. In addition, the putative β-CF_3_ carbocation intermediate formed through SET photoredox processes is playing a key role in our reaction systems (vide infra).

**Scheme 1:**
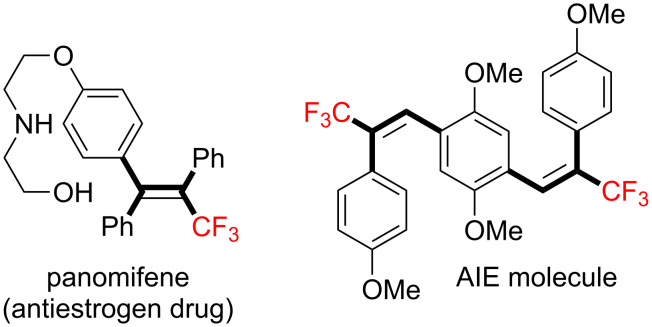
Representative examples of multisubstituted CF_3_-alkenes.

Trifluoromethylated alkenes, especially multi-substituted CF_3_-alkenes (3,3,3-trifluoropropene derivatives), have attracted our attention as fascinating scaffolds for agrochemicals, pharmaceuticals, and fluorescent molecules (Scheme 1) [3,42–45].

**Scheme 2:**
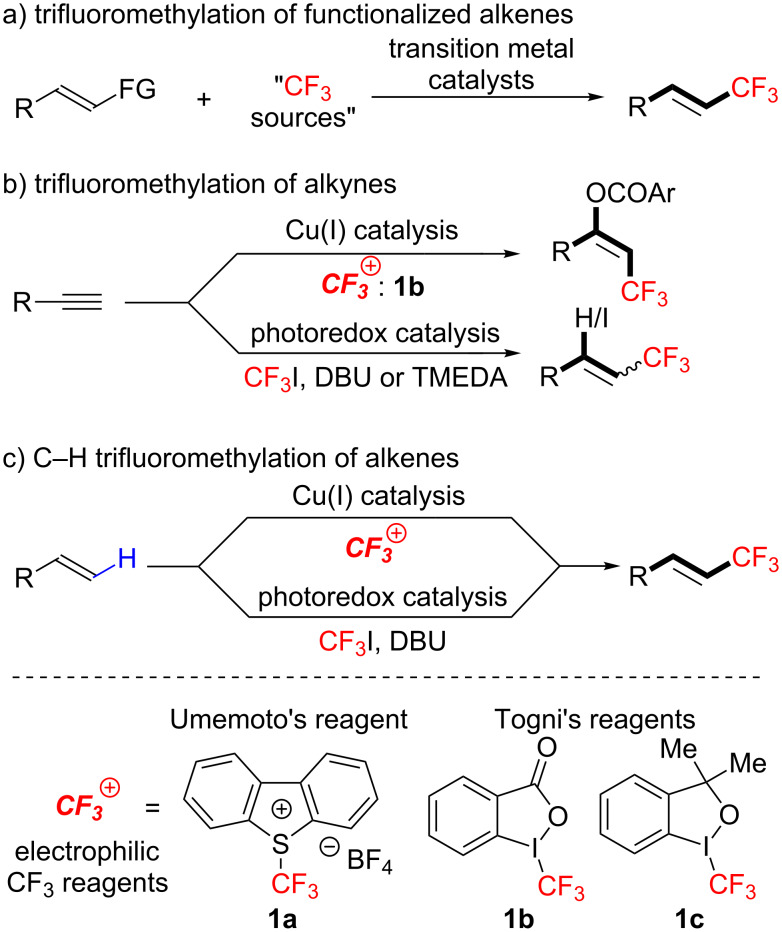
Catalytic synthesis of CF_3_-alkenes via trifluoromethylation.

Conventional approaches to CF_3_-alkenes require multiple synthetic steps [46–54]. In contrast, “trifluoromethylation” is a promising protocol to obtain diverse CF_3_-alkenes easily. Several catalytic synthetic methods via trifluoromethylation have been developed so far [38,55–62]. Most of these reactions require prefunctionalized alkenes as a substrate (Scheme 2a). Additionally, only a limited number of examples for synthesis of tri/tetra-substituted CF_3_-alkenes have been reported so far. Recently, the groups of Szabó and Cho described trifluoromethylation of alkynes, leading to trifluoromethylated alkenes but the application to the synthesis of tetrasubstituted CF_3_-alkenes is not well documented (Scheme 2b) [63–64]. Another straightforward approach is direct C–H trifluoromethylation of alkenes (Scheme 2c). The groups of Loh, Besset, Cahard, Sodeoka and Xiao showed that copper catalysts can induce a C–H trifluoromethylation of alkenes by electrophilic CF_3_ reagents (***^+^CF_3_***) [65–69]. In addition, Cho et al. reported that the reaction of unactivated alkenes with gaseous CF_3_I in the presence of a Ru photocatalyst, [Ru(bpy)_3_]^2+^, and a base, DBU (diazabicyclo[5,4,0]undec-7-ene) produced CF_3_-alkenes through iodotrifluoromethylation of alkenes followed by base-induced E2 elimination [70]. To the best of our knowledge, however, the development of synthetic methods for tri- and tetrasubstituted CF_3_ alkenes through C_alkenyl_–H trifluoromethylation of simple alkenes have been left much to be desired.

**Scheme 3:**
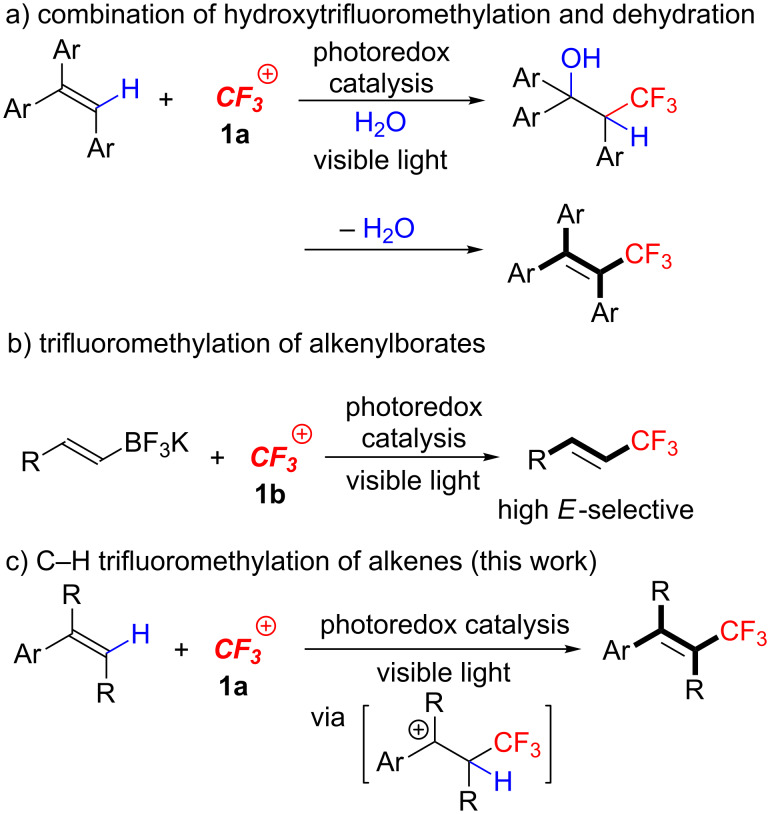
Our strategies for synthesis of CF_3_-alkenes.

Previously, we reported on the synthesis of CF_3_-alkenes via sequential photoredox-catalyzed hydroxytrifluoromethylation and dehydration (Scheme 3a) [37] and photoredox-catalyzed trifluoromethylation of alkenylborates (Scheme 3b) [38]. These results prompted us to explore photoredox-catalyzed C–H trifluoromethylation of di- and trisubstituted alkenes (Scheme 3c). Herein we disclose a highly efficient direct C–H trifluoromethylation of di- and trisubstituted alkenes with easy-handling and shelf-stable Umemoto’s reagent **1a** by visible-light-driven photoredox catalysis under mild conditions. This photocatalytic protocol allows us easy access to a range of multi-substituted trifluoromethylated alkenes. In addition, our methodology can be extended to a double trifluoromethylation of 1,1-disubstituted alkenes.

## Results and Discussion

The results of investigations on the reaction conditions are summarized in Table 1. We commenced examination of photocatalytic trifluoromethylation of 1,1-diphenylethene **2a** with 1 equivalent of Umemoto’s reagent **1a** in the presence of 5 mol % *fac*-Ir(ppy)_3_, a photoredox catalyst, and 2 equivalents of K_2_HPO_4_, a base, in [D_6_]-DMSO under visible light irradiation (blue LEDs: λ_max_ = 425 nm) for 2 h. As a result, 3,3,3-trifluoro-1,1-diphenylpropene (**3a**) was obtained in an 82% NMR yield (Table 1, entry 1). The choice of ***^+^CF_3_*** reagents turned out to be crucial for the yield of **3a**. Togni’s reagents **1b** and **1c** gave **3a** in lower yields (Table 1, entries 2 and 3). We also found that DMSO is suitable for the present reaction (Table 1, entries 4–6). Other solvent systems gave substantial amounts of the hydroxytrifluoromethylated byproduct, which we reported previously [37]. In addition, the present C–H trifluoromethylation proceeds even in the absence of a base (Table 1, entry 7). Another photocatalyst, [Ru(bpy)_3_](PF_6_)_2_, also promoted the present reaction, providing the product **3a** in an 85% NMR yield (Table 1, entry 8). The Ru catalyst is less expensive than the Ir catalyst; thus, we chose the Ru photocatalyst for the experiments onward. Notably, product **3a** was obtained neither in the dark nor in the absence of photocatalyst (Table 1, entries 9 and 10), strongly supporting that the photoexcited species of the photoredox catalyst play key roles in the reaction.

**Table 1:** Optimization of photocatalytic trifluoromethylation of 1,1-diphenylethene **2a**.^a^

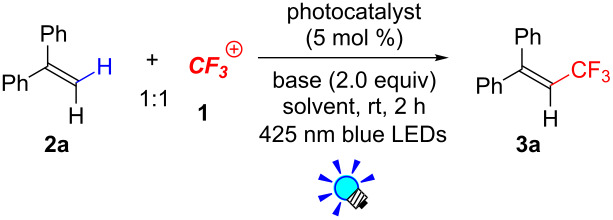					

Entry	Photocatalyst	CF_3_ reagent	Solvent	Base	NMR yield (%)

1	*fac*-Ir(ppy)_3_	**1a**	[D_6_]-DMSO	K_2_HPO_4_	82
2	*fac*-Ir(ppy)_3_	**1b**	[D_6_]-DMSO	K_2_HPO_4_	17
3	*fac*-Ir(ppy)_3_	**1c**	[D_6_]-DMSO	K_2_HPO_4_	47
4	*fac*-Ir(ppy)_3_	**1a**	CD_3_CN	K_2_HPO_4_	57
5	*fac*-Ir(ppy)_3_	**1a**	CD_2_Cl_2_	K_2_HPO_4_	22
6	*fac*-Ir(ppy)_3_	**1a**	[D_6_]-acetone	K_2_HPO_4_	29
7	*fac*-Ir(ppy)_3_	**1a**	[D_6_]-DMSO	none	81
8	[Ru(bpy)_3_](PF_6_)_2_	**1a**	[D_6_]-DMSO	none	85
9	none	**1a**	[D_6_]-DMSO	none	0
10^b^	[Ru(bpy)_3_](PF_6_)_2_	**1a**	[D_6_]-DMSO	none	0

^a^For reaction conditions, see the Experimental section. ^b^In the dark.

The scope and limitations of the present photocatalytic trifluoromethylation of alkenes are summarized in Table 2. 1,1-Diphenylethenes with electron-donating substituents, MeO (**2b**), and halogens, Cl (**2c**) and Br (**2d**), smoothly produced the corresponding trisubstituted CF_3_-alkenes (**3b**–**d**) in good yields. In the reactions of unsymmetrically substituted substrates (**2e**–**h**), products were obtained in good to moderate yields but consisted of mixtures of *E* and *Z-*isomers. Based on the experimental results, the *E*/*Z* ratios are susceptible to the electronic structure of the aryl substituent. Reactions afforded the major isomers, in which the CF_3_ group and the electron-rich aryl substituent are arranged in *E*-fashion. In addition, the present photocatalytic reaction can be tolerant of the Boc-protected amino group (**2f**) or pyridine (**2h**). Moreover, a substrate with an alkyl substituent, 2,4-diphenyl-4-methyl-1-pentene (**2i**), was also applicable to this transformation, whereas the reaction of 1,2-disubsituted alkenes such as *trans*-stilbene provided complicated mixtures of products.

**Table 2:** The scope of the present trifluoromethylation of alkenes.^a, b^

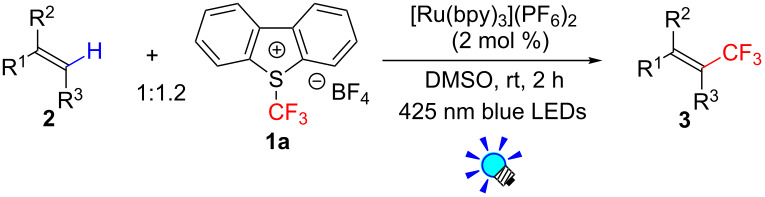				

Trifluoromethylated products **3a–m**				

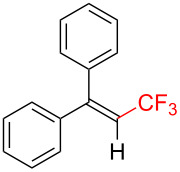	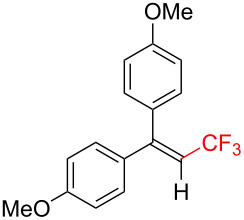	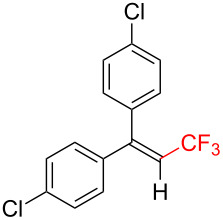	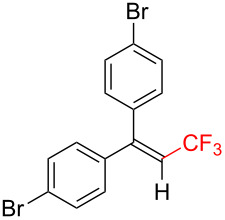	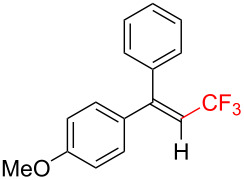
**3a**: 82%^c^	**3b**: 81%^c^	**3c**: 53%	**3d**: 70%	**3e**: 70%^c^ *E*/*Z* = 89/11^d^
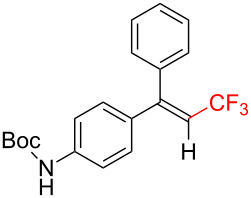	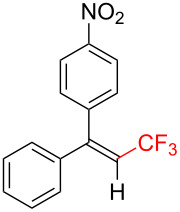	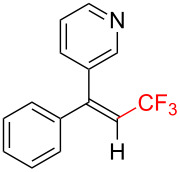	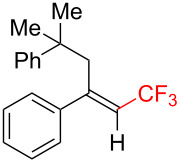	
**3f**: 37%^e^ *E*/*Z* = 91/9^d^	**3g**: 51% *E*/*Z* = 17/83^d^	**3h**: 78% *E*/*Z* = 33/67^d^	**3i**: 58% *E*/*Z* = 88/12^d^	
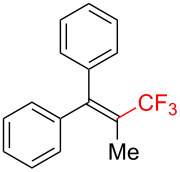	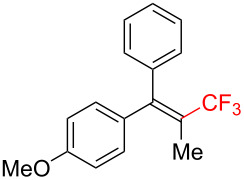	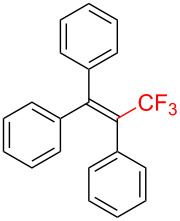	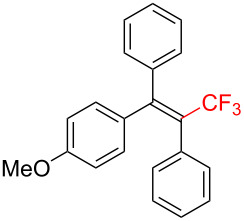	
**3j**: 82%	**3k**: 59% *E*/*Z* = 74/26^d^	**3l**: 65%	**3m**: 53% *E*/*Z* = 88/12^d^	

^a^For reaction conditions, see the Experimental section. ^b^Isolated yields. ^c^NMR yields. ^d^*E*/*Z* ratios were determined by ^19^F NMR spectroscopy of the crude product mixtures. ^e^2,6-Lutidine (2 equiv) was added as a base.

Next, we extended the present C–H trifluoromethylation to trisubstituted alkenes. The reactions of 1,1-diphenylpropene derivatives **2j** and **2k** (*E*/*Z* = 1/1) afforded the corresponding tetrasubstituted CF_3_-alkenes **3j** and **3k** in 82% and 59% (*E*/*Z* = 74/26) yields, respectively. Triphenylethenes **2l** and **2m** (only *E-*isomer) are also applicable to this photocatalytic C–H trifluoromethylation. Remarkably, the *E-*isomer of **3m** is a key intermediate for the synthesis of panomifene, which is known as an antiestrogen drug [71–72]. These results show that the present protocol enables the efficient construction of a C_alkenyl_–CF_3_ bond through direct C–H trifluoromethylation of 1,1-disubstituted and trisubstituted aryl alkenes.

**Scheme 4:**
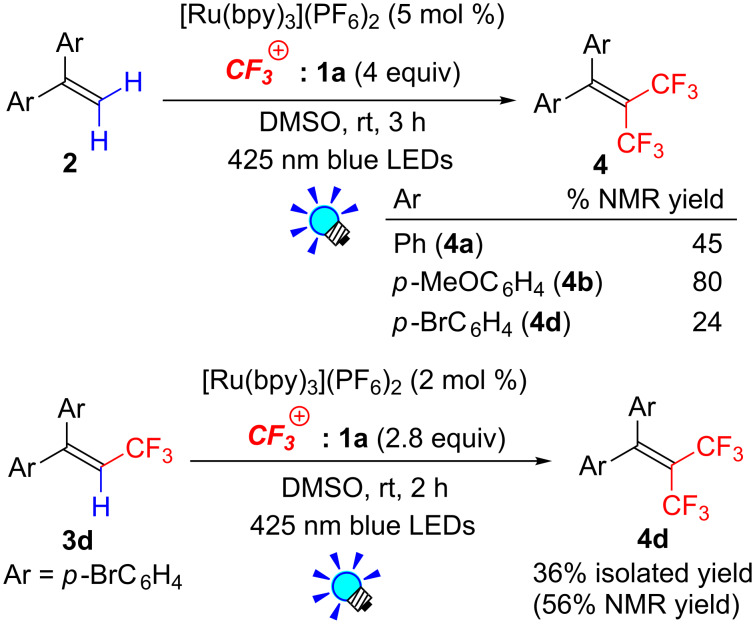
Synthesis of geminal bis(trifluoromethyl)alkenes.

During the course of our study on the C–H trifluoromethylation of 1,1-diarylethenes **2**, we found that a detectable amount of bis(trifluoromethyl)alkenes **4** was formed through double C–H trifluoromethylation. In fact, the photocatalytic trifluoromethylation of **2a**,**b** and **d** with 4 equivalents of Umemoto’s reagent **1a** in the presence of 5 mol % of [Ru(bpy)_3_](PF_6_)_2_ with irradiation from blue LEDs for 3 h gave geminal bis(trifluoromethyl)ethene (**4a**,**b** and **d**) in 45, 80 and 24% NMR yields, respectively (Scheme 4). Substituents on the benzene ring significantly affect the present double trifluoromethylation. Reaction of the electron-rich alkene **2b** afforded 1,1-anisyl-2,2-bis(trifluoromethyl)ethene (**4b**) in a better yield than other alkenes **2a** and **2d**. Additionally, we found that photocatalytic trifluoromethylation of CF_3_-alkene **3d** in the presence of an excess amount of Umemoto’s reagent **1a** produced bis(trifluoromethyl)alkenes **4d** in a better yield (56% yield) compared to the above-mentioned one-pot double trifluoromethylation of **2d**.

**Scheme 5:**
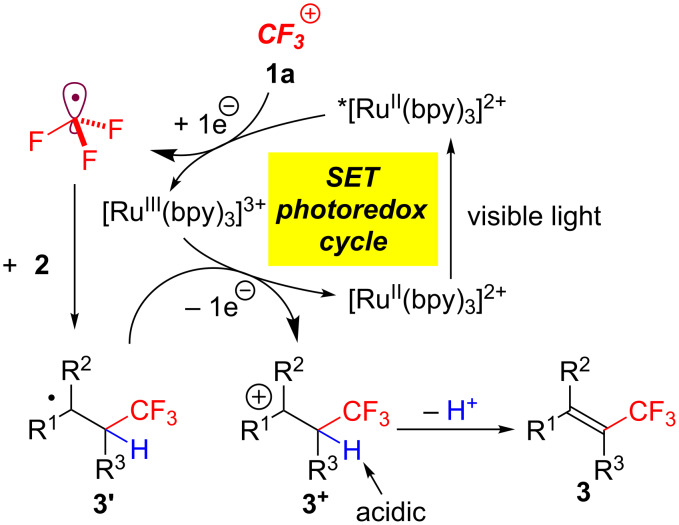
A possible reaction mechanism.

A possible reaction mechanism based on SET photoredox processes is illustrated in Scheme 5. According to our previous photocatalytic trifluoromethylation [37–41], the trifluoromethyl radical (·CF_3_) is generated from an one-electron-reduction of electrophilic Umemoto’s reagent **1a** by the photoactivated Ru catalyst, *[Ru(bpy)_3_]^2+^. ·CF_3_ reacts with alkene **2** to give the benzyl radical-type intermediate **3'** in a regioselective manner. Subsequent one-electron-oxidation by highly oxidizing Ru species, [Ru^III^(bpy)_3_]^3+^, produces β-CF_3_ carbocation intermediate **3^+^**. Finally, smooth elimination of the olefinic proton, which is made acidic by the strongly electron-withdrawing CF_3_ substituent, provides trifluoromethylated alkene **3**. Preferential formation of one isomer in the reaction of unsymmetrical substrates is attributed to the population of the rotational conformers of the β-CF_3_ carbocation intermediate **3^+^**. Our experimental result is consistent with the previous report [71], which described *E*-selective formation of the tetrasubstituted CF_3_-alkene **3m** via a β-CF_3_ carbocation intermediate. In the presence of an excess amount of CF_3_ reagent **1a**, further C–H trifluoromethylation of CF_3_-alkene **3** proceeds to give bis(trifluoromethyl)alkene **4**.

**Figure 1:**
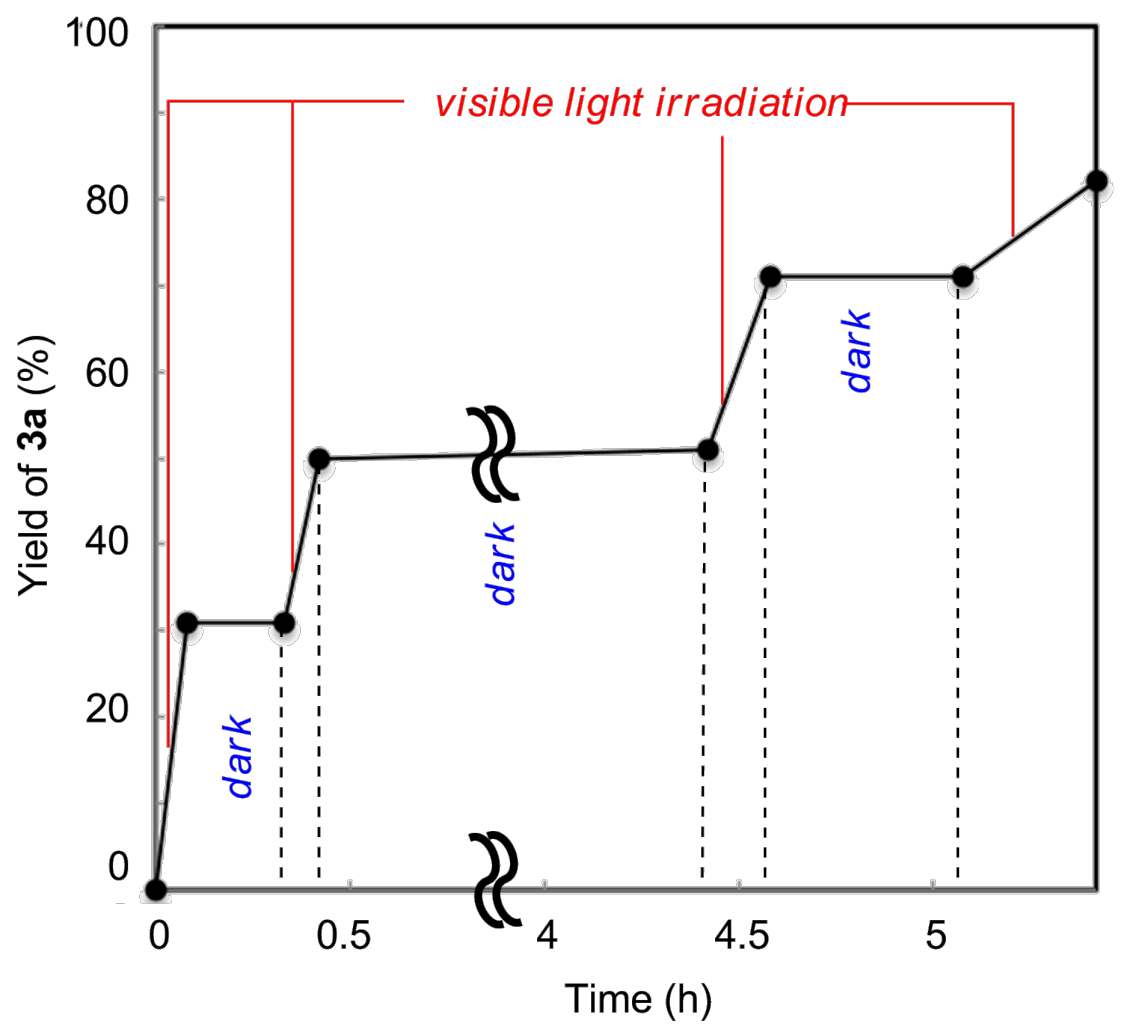
Time profile of the photocatalytic trifluoromethylation of **2a** with **1a** with intermittent irradiation by blue LEDs.

We cannot rule out a radical chain propagation mechanism, but the present transformation requires continuous irradiation of visible light (Figure 1), thus suggesting that chain propagation is not a main mechanistic component.

## Conclusion

We have developed highly efficient C–H trifluoromethylation of alkenes using Umemoto’s reagent as a CF_3_ source by visible-light-driven photoredox catalysis. This reaction can be applied to multi-substituted alkenes, especially, 1,1-disubstituted and trisubstituted aryl alkenes, leading to tri- and tetrasubstituted CF_3_-alkenes. The present straightforward method for the synthesis of multisubstituted CF_3_-alkenes from simple aryl alkenes is the first report. In addition, we can extend the present photocatalytic system to double trifluoromethylation. Further development of this protocol in the synthesis of bioactive organofluorine molecules and fluorescent molecules is a continuing effort in our laboratory.

## Experimental

### Typical NMR experimental procedure (reaction conditions in Table 1)

Under N_2_, [Ru(bpy)_3_](PF_6_)_2_ (1.1 mg, 1.3 μmol), Umemoto’s reagent **1a** (8.5 mg, 25 μmol), 1,1-diphenylethylene (**2a**, 4.3 μL, 25 μmol), SiEt_4_ (~1 μL) as an internal standard, and [D_6_]-DMSO (0.5 mL) were added to an NMR tube. The reaction was carried out at room temperature (water bath) under irradiation of visible light (placed at a distance of 2–3 cm from 3 W blue LED lamps: *h*ν = 425 ± 15 nm).

### General procedure for the photocatalytic C−H trifluoromethylation of alkenes (reaction conditions in Table 2)

A 20 mL Schlenk tube was charged with Umemoto’s reagent **1a** (102 mg, 0.3 mmol, 1.2 equiv), [Ru(bpy)_3_](PF_6_)_2_ (4.3 mg, 2 mol %), alkene **2** (0.25 mmol), and DMSO (2.5 mL) under N_2_. The tube was irradiated for 2 h at room temperature (water bath) with stirring by 3 W blue LED lamps (*h*ν = 425 ± 15 nm) placed at a distance of 2–3 cm. After the reaction, H_2_O was added. The resulting mixture was extracted with Et_2_O, washed with H_2_O, dried (Na_2_SO_4_), and filtered. The filtrate was concentrated in vacuo*.* The product was purified by the two methods described below.

For products **3b**, **3e**, **3f**, **3g**, **3h**, **3k** and **3m**, the residue was purified by column chromatography on silica gel (eluent: hexane and diethyl ether) to afford the corresponding product **3**. Further purification of **3f** by GPC provided pure **3f**. For products **3a**, **3c**, **3d**, **3i**, **3j**, and **3l**, the residue was treated by mCPBA (74 mg, ca. 0.3 mmol) in CH_2_Cl_2_ to convert the dibenzothiophene to sulfoxide, which was more easily separated from the products. After the solution was stirred at room temperature for 2 h, an aqueous solution of Na_2_S_2_O_3_·5H_2_O was added to the solution, which was extracted with CH_2_Cl_2_. The organic layer was washed with H_2_O, dried (Na_2_SO_4_), and filtered. The filtrate was concentrated in vacuo and the residue was purified by flash column chromatography on silica gel (eluent: hexane) to afford the corresponding product **3**. Further purification of **3c** and **3d** by GPC provided pure **3c** and **3d**.

### Procedures for the photocatalytic double C−H trifluoromethylation of 1,1-bis(4-methoxyphenyl)ethylene (**2b**)

A 20 mL Schlenk tube was charged with Umemoto’s reagent **1a** (340 mg, 1.0 mmol, 4 equiv), [Ru(bpy)_3_](PF_6_)_2_ (10.7 mg, 5 mol %), **2b** (60 mg, 0.25 mmol), and DMSO (5 mL) under N_2_. The tube was irradiated for 3 h at room temperature (water bath) with stirring by 3 W blue LED lamps (*h*ν = 425 ± 15 nm) placed at a distance of 2–3 cm. After reaction, H_2_O was added. The resulting mixture was extracted with Et_2_O, washed with H_2_O, dried (Na_2_SO_4_), and filtered. The filtrate was concentrated in vacuo and the residue was purified by flash column chromatography on silica gel (hexane→hexane/Et_2_O = 29:1) to afford **4b** as a product mixture with **3b**. Further purification by GPC provided pure **4b** in 44% isolated yield (42 mg, 0.11 mmol). Isolated yield was much lower than the NMR yield because of the difficulty of separation of **3b** and **4b**.

## Supporting Information

Supporting information features experimental procedures and full spectroscopic data for all new compounds (**3c**, **3d**, **3f**, **3g**, **3h**, **3i**, **3k**, **4a**, and **4d**).

File 1 Experimental procedures and NMR spectra.
