# Supplementary material for: Direct C–H trifluoromethylation of di- and trisubstituted alkenes by photoredox catalysis
Source: Beilstein J Org Chem. 2014 May 12;10:1099–106. doi: 10.3762/bjoc.10.108 (PMC4077392; doi:10.3762/bjoc.10.108)
Supplement: File 1 — Experimental procedures and NMR spectra. [file Beilstein_J_Org_Chem-10-1099-s001.pdf]

Supporting Information

for

Direct C–H trifluoromethylation of di- and trisubstituted alkenes by photoredox catalysis

Ren Tomita, Yusuke Yasu, Takashi Koike* and Munetaka Akita*

Address: Chemical Resources Laboratory, Tokyo Institute of Technology, R1-27, 4259 Nagatsuta, Midori-ku, Yokohama 226-8503, Japan

Email: Takashi Koike - koike.t.ad@m.titech.ac.jp; Munetaka Akita - makita@res.titech.ac.jp

*Corresponding author

Experimental procedures and NMR spectra

Contents

•Materials and methods······ S2
•Reaction apparatus······ S2
•Synthesis of alkene **2**······ S3
•Typical NMR experimental procedures and NMR spectra······ S4
•General procedure for the photocatalytic C–H trifluoromethylation of alkenes (Table 2)······ S5
•Procedures for the photocatalytic double C–H trifluoromethylation of alkenes (Scheme 4)··· S10
•Time profile of the photocatalytic C–H trifluoromethylation of **2a** (Figure 1)······ S12
•Assignment of stereochemistry of product **3**······ S13
•References······ S17
•^{1}H, ^{13}C NMR spectra······ S18

Materials and methods

$[Ru(bpy)_3](PF_6)_2$[1] and *fac*-$Ir(ppy)_3$[2] were prepared according to the literature procedures. Umemoto's reagent (**1a**) was purchased from Aldrich. Alkenes **2b**, **2c**, **2d**, **2e**, **2f**, **2g**, **2h**, **2j**, and **2k** were prepared by Wittig reactions. (*E*)-1,2-diphenyl-1-(4-methoxyphenyl)ethylene (**2m**) prepared according to the literature procedure[3]. Catalytic reactions were performed under an atmosphere of nitrogen using standard Schlenk techniques unless otherwise noted. All solvents were dried over molecular sieves, degassed and stored under N_2. Thin-layer chromatography was performed on Merck TLC plate with 60 F_{254}. Visible light irradiations were performed with a Relyon LED lamp (3 W x 2; λ_{max} = 425 ± 15 nm). Japan Analytical Industry LC-9201 was utilized for recycling preparative HPLC (GPC). The ^{1}H NMR was acquired on Bruker AVANCE-400 (400 MHz). NMR chemical shifts were referenced to residual protio impurities in the deuterated solvent. HRMS (ESI-TOF mass spectra) were obtained with a Bruker micrOTOF II.

Reaction apparatus

Irradiation of visible light was performed with a Relyon LED lamp (3 W x 2; λ_{max} = 425 nm).

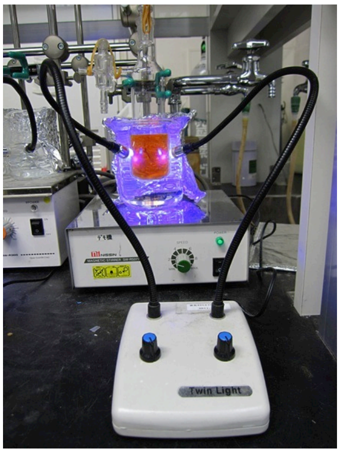

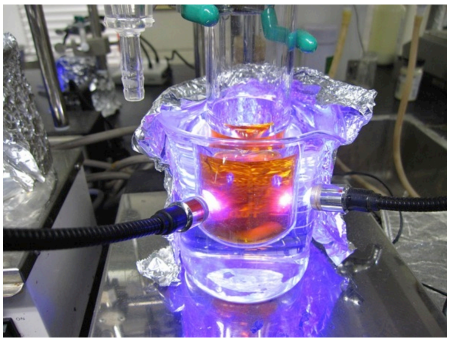

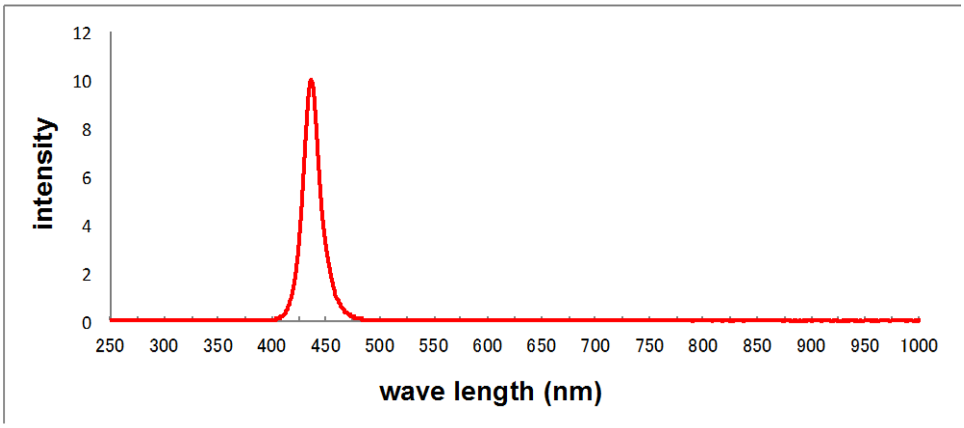

Figure S1: The emission spectrum of a Relyon LED lamp.

Synthesis of alkene 2

General procedure for the synthesis of alkenes (2b, 2c, 2d, 2e, 2g, 2h, 2j and 2k) by Wittig reaction

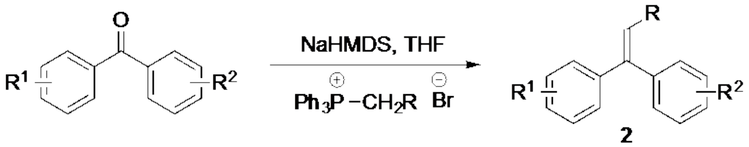

Under N_2, a 2-neck 100 mL round-bottom flask was charged with Wittig reagent (6 mmol) and dry THF (25 mL). Then sodium hexamethyldisilazide (1 M THF solution, 6 mL) was added into the solution and stirred at room temperature for 1 h. To the solution, benzophenone derivative (5 mmol) was added and stirred at room temperature overnight. Et_2O was added into the reaction mixture and filtered. The filtrate was concentrated in vacuo and the residue was purified by flash column chromatography on silica gel to afford the product.

The synthesis of *N*-(*tert*-butoxycarbonyl)-4-(1-phenylvinyl)aniline (2f)

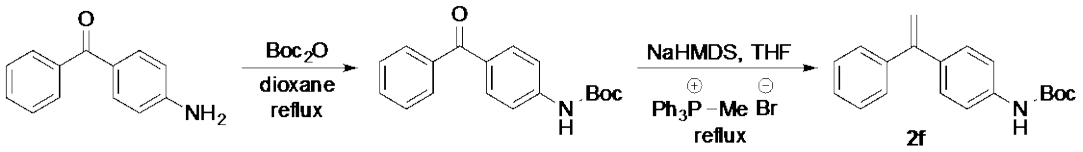

***N*-(*tert*-Butoxycarbonyl)-4-benzoylaniline:** A 2-neck 100 mL round-bottom flask was charged with 4-aminobenzophenone (1.97 g, 10 mmol), di-*tert*-butyl dicarbonate (2.84 g, 13 mmol), and 1,4-dioxane (30 mL). The solution was refluxed overnight and cooled to room temperature. The reaction mixture was concentrated in vacuo and the residue was purified by flash column chromatography on silica gel (hexane/EtOAc = 4:1) to afford *N*-(*tert*-butoxycarbonyl)-4-benzoylaniline (1.35 g, 46% yield).

***N*-(*tert*-Butoxycarbonyl)-4-(1-phenylvinyl)aniline (2f):** Under N_2, a 2-neck 100 mL round-bottom flask was charged with methyltriphenylphosphonium bromide (1.07 g, 3 mmol) and dry THF (15 mL). Then sodium hexamethyldisilazide (1 M THF solution, 3 mL) was added into the solution and stirred at room temperature for 1 h. To the yellow solution, *N*-(*tert*-butoxycarbonyl)-4-benzoylaniline (0.53 g, 1.8 mmol) was added and refluxed overnight. After cooling to room temperature, Et_2O was added into the reaction mixture and filtered. The filtrate was concentrated in vacuo and the residue was purified by flash column chromatography on silica gel (hexane/EtOAc = 4:1) to afford *N*-(*tert*-butoxycarbonyl)-4-(1-phenylvinyl)aniline (0.23 g, 44% yield).

^{1}H NMR (400 MHz, $CDCl_3$, rt): δ 7.34-7.26 (m, 9 H, Ar), 6.48 (brs, 1 H, N*H*), 5.41 (d, *J* = 1.2 Hz, C=C*H*H), 5.38 (d, *J* = 1.2 Hz, C=CH*H*), 1.53 (s, 9 H, C(CH_3)$_3$). **^{13}C NMR** (100 MHz, $CDCl_3$, rt): δ 152.8, 149.6, 141.7, 138.1, 136.4, 129.0, 128.4, 128.3, 127.8, 118.3, 113.6, 80.7,

28.5. **HRMS** (ESI-TOF): calculated for $[C_{19}H_{21}NO_2+Na]^+$ requires 318.1465, found 318.1464.

Typical NMR experimental procedures and NMR spectra

NMR experimental procedure (entry 1 in Table 1)

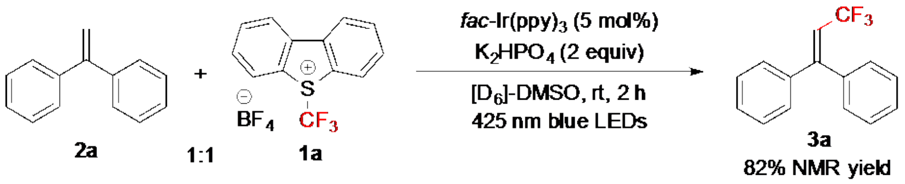

Under N_2, *fac*-Ir(ppy)$_3$ (0.8 mg, 1.3 µmol), Umemoto's reagent (**1a**, 8.5 mg, 25 µmol), K_2HPO_4 (8.7 mg, 50 µmol), 1,1-diphenylethylene (**2a**, 4.3 µL, 25 µmol), $SiEt_4$ (~1 µL) as an internal standard, and [D_6]-DMSO (0.5 mL) were added to an NMR tube. The reaction was carried out at room temperature (water bath) under irradiation of visible light (placed at a distance of ~3 cm from a blue LED lamp: $h\nu = 425 \pm 15$ nm).

NMR experimental procedure (entry 8 in Table 1) and NMR spectra

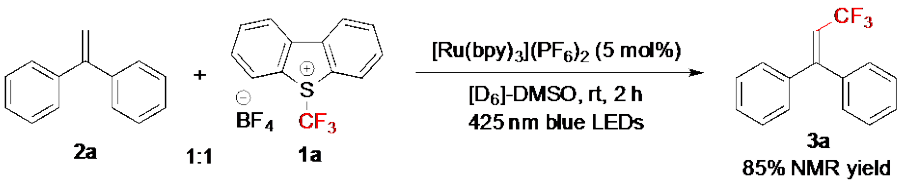

Under N_2, [Ru(bpy)$_3$](PF$_6$)$_2$ (1.1 mg, 1.3 µmol), Umemoto's reagent (**1a**) (8.5 mg, 25 µmol), 1,1-diphenylethylene (**2a**) (4.3 µL, 25 µmol), $SiEt_4$ (~1 µL) as an internal standard, and [D_6]-DMSO (0.5 mL) were added to an NMR tube. The reaction was carried out at room temperature (water bath) under irradiation of visible light (placed at a distance of ~3 cm from blue LED lamp: $h\nu = 425 \pm 15$ nm).

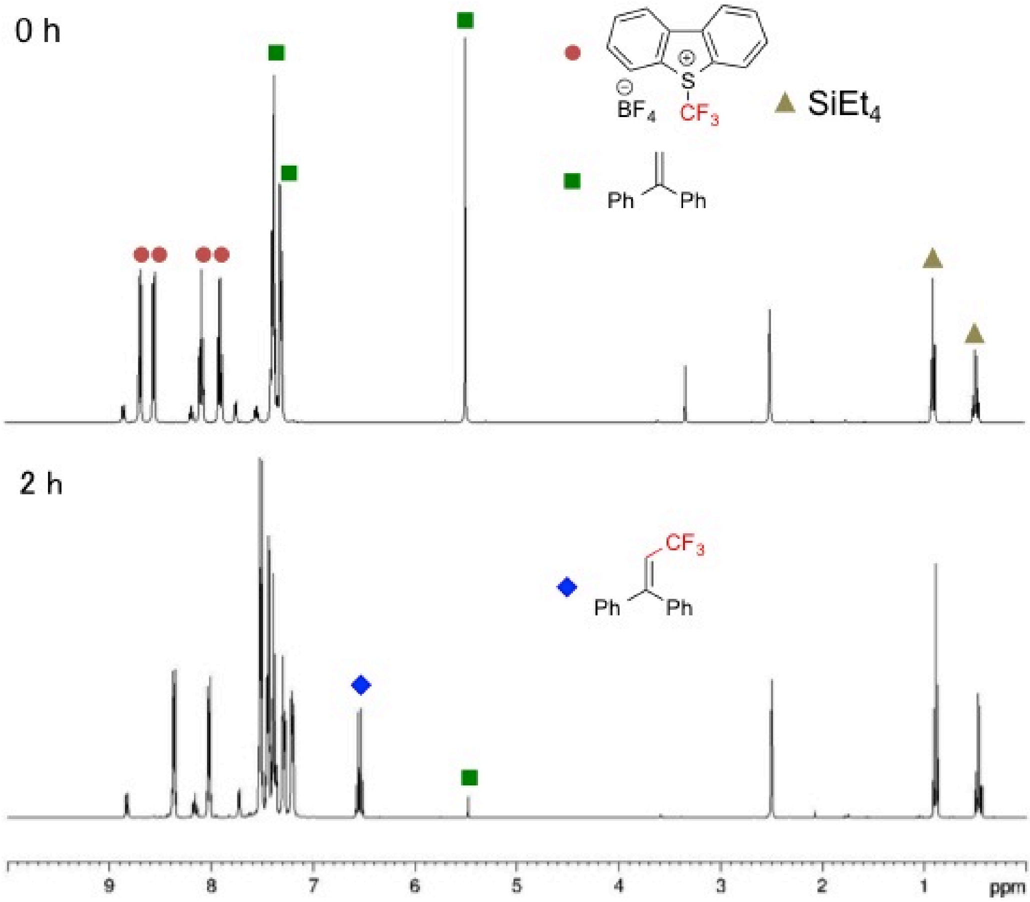

Figure S2. ^{1}H NMR spectra (400 MHz, [D$_6$]-DMSO, rt)

General procedure for the photocatalytic C–H trifluoromethylation of alkenes (Table 2, 3a–e, g–m)

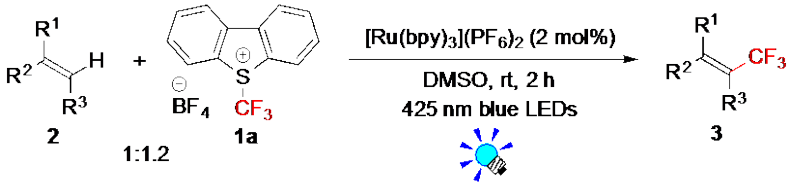

A 20 mL-Schlenk tube was charged with Umemoto's reagent (**1a**, 102 mg, 0.3 mmol, 1.2 equiv.), [Ru(bpy)$_3$](PF$_6$)$_2$ (4.3 mg, 2 mol %), alkene **2** (0.25 mmol), and DMSO (2.5 mL) under N_2. The tube was irradiated for 2 h at room temperature (water bath) with stirring by 3 W blue LED lamps ($h\nu$ = 425 nm ± 15 nm) placed at a distance of 2–3 cm. After the reaction, H_2O was added. The resulting mixture was extracted with Et_2O, washed with H_2O, dried (Na_2SO_4), and filtered. The filtrate was concentrated in vacuo. The product was purified in two ways as described below.

For products **3b**, **3e**, **3g**, **3h**, **3k** and **3m**, the residue was purified by flash column chromatography on silica gel (eluent: hexane and Et_2O) to afford the corresponding product **3**.

For products **3a**, **3c**, **3d**, **3i**, **3j** and **3l**, the residue was treated by mCPBA (74 mg, ca. 0.3 mmol)

in CH_2Cl_2 to convert the dibenzothiophene to sulfoxide, which was more easily separated from the products. After the solution was stirred at room temperature for 2 h, an aqueous solution of $Na_2S_2O_3 \cdot 5H_2O$ was added to the solution, which was extracted with CH_2Cl_2. The organic layer was washed with H_2O, dried (Na_2SO_4), and filtered. The filtrate was concentrated in vacuo and the residue was purified by flash column chromatography on silica gel (eluent: hexane) to afford the corresponding product **3**. Further purification of **3c** and **3d** by GPC provided pure **3c** and **3d**. **3a**[4], **3b**[5], **3e**[5], **3j**[6], **3l**[7] and **3m**[7] were confirmed by published data.

1,1-Bis(4-chlorophenyl)-3,3,3-trifluoropropene (3c)

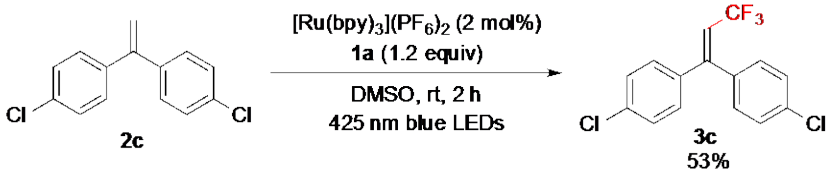

According to the general procedure, the title compound was synthesized from 1,1-bis(4-chlorophenyl)ethylene (**2c**, 62 mg, 0.25 mmol). The product was purified by silica gel flash column chromatography (hexane) to afford **3c** (82%) as a product mixture with bis(trifluoromethyl)alkene **4c** (8%). The yields were determined by ^{19}F NMR using α,α,α-trifluorotoluene as an internal standard. Further purification by GPC provided pure **3c** in 53% isolated yield.

^{1}H NMR (400 MHz, $CDCl_3$, rt): δ 7.38 (d, J = 8.8 Hz, 2 H, Ar), 7.32 (d, J = 8.8 Hz, 2 H, Ar), 7.16 (d, J = 8.4 Hz, 4 H, Ar), 6.12 (q, J = 8.1 Hz, 1 H, C=C*H*CF_3). **^{13}C NMR** (100 MHz, $CDCl_3$, rt): δ 150.4 (q, J = 5.4 Hz), 138.3, 136.1, 135.3, 135.2, 130.6, 129.3, 129.0, 128.7, 122.9 (q, J = 269 Hz), 116.5 (q, J = 33.9 Hz). **^{19}F NMR** (376.5 MHz, $CDCl_3$, rt): δ -55.76 (d, J = 7.91 Hz, 3 F). **HRMS** (ESI-TOF): calculated for $[C_{15}H_9Cl_2F_3+Na]^+$ requires 338.9926, found 338.9967.

1,1-Bis(4-bromophenyl)-3,3,3-trifluoropropene (3d)

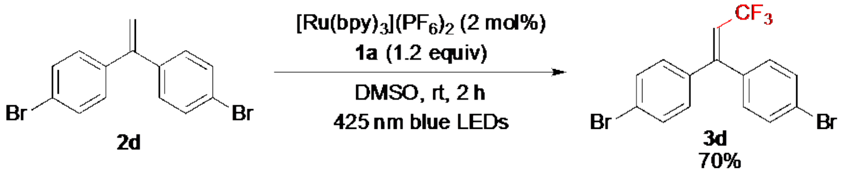

According to the general procedure, the title compound was synthesized from 1,1-bis(4-bromophenyl)ethylene (**2d**, 85 mg, 0.25 mmol). The product was purified by silica gel flash column chromatography (hexane) to afford **3d** (84%) as a product mixture with bis(trifluoromethyl)alkene **4d** (6%). The yields were determined by ^{19}F NMR using α,α,α-trifluorotoluene as an internal standard. Further purification by GPC provided pure **3d** in

70% isolated yield.

^{1}H NMR (400 MHz, $CDCl_3$, rt): δ 7.54 (d, J = 8.4 Hz, 2 H, Ar), 7.47 (d, J = 8.4 Hz, 2 H, Ar), 7.10 (d, J = 8.4 Hz, 4 H, Ar), 6.12 (q, J = 8.1 Hz, 1 H, C=C*H*CF_3). **^{13}C NMR** (100 MHz, $CDCl_3$, rt): δ 150.4 (q, J = 5.4 Hz), 138.6, 135.7, 132.0, 131.7, 130.9, 129.6, 124.3, 123.4, 122.9 (q, J = 269 Hz), 116.5 (q, J = 34.0 Hz). **^{19}F NMR** (376.5 MHz, $CDCl_3$, rt): δ -55.72 (d, J = 7.91 Hz, 3 F). **HRMS** (ESI-TOF): calculated for $[C_{15}H_9Br_2F_3+Na]^+$ requires 426.8915, found 426.9055.

N-(*tert*-Butoxycarbonyl)-4-(1-phenyl-3,3,3-trifluoropropenyl)aniline (3f)

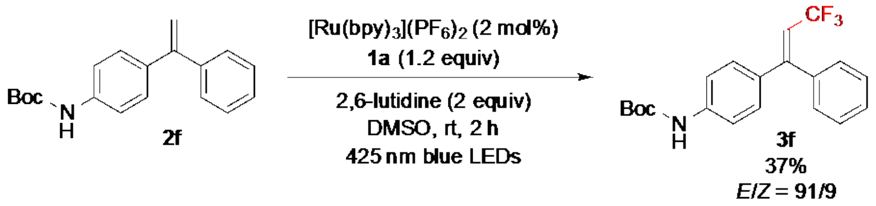

A 20 mL-Schlenk tube was charged with Umemoto's reagent (**1a**, 102 mg, 0.3 mmol, 1.2 equiv.), $[Ru(bpy)_3](PF_6)_2$ (4.3 mg, 2 mol %), *N*-(*tert*-butoxycarbonyl)-4-(1-phenylvinyl)aniline (**2f**, 74 mg, 0.25 mmol), 2,6-lutidine (58 μL, 0.5 mmol, 2 equiv) and DMSO (2.5 mL) under N_2. The tube was irradiated for 2 h at room temperature (water bath) with stirring by 3 W blue LED lamps ($h\nu$ = 425 nm ± 15 nm) placed at a distance of 2–3 cm. After the reaction, H_2O was added. The resulting mixture was extracted with Et_2O, washed with H_2O, dried (Na_2SO_4), and filtered. The filtrate was concentrated in vacuo. The residue was purified by flash column chromatography on silica gel (hexane→hexane/EtOAc = 9:1) to afford **3f** (60 mg, 59% yield, *E*/*Z* = 91:9) as a product mixture with bis(trifluoromethyl)alkene **4f** (21%). The yields were determined by ^{19}F NMR using α,α,α-trifluorotoluene as an internal standard. Further purification by GPC provided pure **3f** in 37% isolated yield. The stereochemistry was confirmed by ^{1}H NOESY NMR.

^{1}H NMR (400 MHz, $CDCl_3$, rt): δ 7.38 (m, 3 H, Ar), 7.32 (d, J = 8.8 Hz, 2 H, Ar), 7.22 (m, 2H, Ar), 7.17 (d, J = 8.4 Hz, 2 H, Ar), 6.53 (brs, 1 H, N*H*), 6.08 (q, J = 8.4 Hz, 1 H, C=C*H*CF_3 in *E*-isomer), 6.06 (q, J = 8.0 Hz, 1 H, C=C*H*CF_3 in *Z*-isomer), 1.53 (s, 9 H, $C(CH_3)_3$ in *Z*-isomer), 1.52 (s, 9 H, $C(CH_3)_3$ in *E*-isomer). **^{13}C NMR** (100 MHz, $CDCl_3$, rt): δ 152.6, 152.0 (q, J = 5.3 Hz), 139.8, 137.5, 134.7, 129.3, 128.9, 128.6, 128.1, 123.4 (q, J = 269 Hz), 118.32, 114.2 (q, J = 33.6 Hz), 81.1, 28.4. **^{19}F NMR** (376.5 MHz, $CDCl_3$, rt): δ -55.32 (d, J = 8.28 Hz, 3 F, *E*-isomer), -55.56 (d, J = 9.04 Hz, 3 F, *Z*-isomer). **HRMS** (ESI-TOF): calculated for $[C_{20}H_{20}F_3NO_2+Na]^+$ requires 386.1338, found 386.1332.

3,3,3-Trifluoro-1-(4-nitrophenyl)-1-phenylpropene (3g)

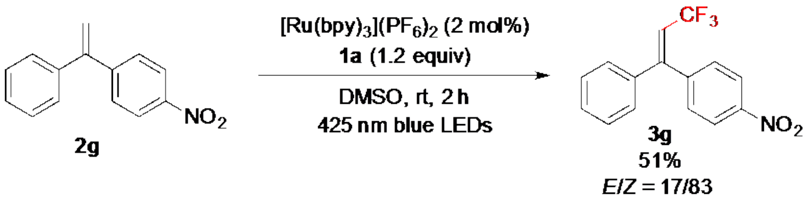

According to the general procedure, the title compound was synthesized from 1-(4-nitrophenyl)-1-phenylethylene (**2g**, 56 mg, 0.25 mmol). The product was purified by silica gel flash column chromatography (hexane→hexane/Et_2O = 19:1) to afford **3g** (39 mg, 51% yield, *E*/*Z* = 17:83). The stereochemistry was confirmed by ^{1}H NOESY NMR.

^{1}**H NMR** (400 MHz, $CDCl_3$, rt): δ 8.27 (d, *J* = 8.8 Hz, 2 H, Ar in *Z-isomer*), 8.19 (d, *J* = 8.8 Hz, 2 H, Ar in *E-isomer*), 7.45-7.35 (m, 5 H, Ar), 7.21 (d, *J* = 8.0 Hz, 2 H, Ar), 6.25 (q, *J* = 8.1 Hz, 1 H, C=C*H*CF_3 in *Z-isomer*), 6.22 (q, *J* = 8.0 Hz, 1 H, C=C*H*CF_3 in *Z-isomer*). ^{13}**C NMR** (100 MHz, $CDCl_3$, rt): δ 150.4 (q, *J* = 5.4 Hz), 148.5, 148.2, 146.4, 144.1, 138.7, 136.1, 130.3, 130.2, 129.3, 129.1, 129.0, 128.6, 127.9, 123.9, 123.5, 122.8 (q, *J* = 269 Hz), 118.6 (q, *J* = 33.8 Hz, *E*-isomer), 117.1 (q, *J* = 34.0 Hz, *Z*-isomer). ^{19}**F NMR** (376.5 MHz, $CDCl_3$, rt): δ -55.72 (d, *J* = 8.28 Hz, 3 F, *Z-isomer*), -56.23 (d, *J* = 8.00 Hz, 3 F, *E-isomer*). **HRMS** (ESI-TOF): calculated for $[C_{15}H_{10}F_3NO_2+Na]^+$ requires 316.0556, found 316.0553.

3,3,3-Trifluoro-1-phenyl-1-(3-pyridyl)propene (3h)

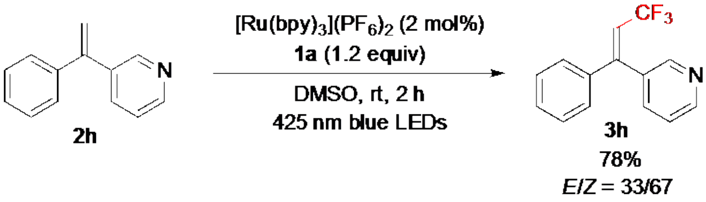

According to the general procedure, the title compound was synthesized from 1-phenyl-1-(3-pyridyl)ethylene (**2h**) (45 mg, 0.25 mmol). The product was purified by silica gel flash column chromatography (hexane→hexane/Et_2O = 1:1) to afford **3h** (48 mg, 78% yield, *E*/*Z* = 33:67). The stereochemistry was confirmed by ^{1}H NOESY NMR.

^{1}**H NMR** (400 MHz, $CDCl_3$, rt): δ 8.66 (dd, *J* = 4.8, 1.6 Hz, 1H, Ar in *Z*-isomer), 8.60 (dd, *J* = 4.8, 1.2 Hz, 1H, Ar in *E-isomer*), 8.56 (d, *J* = 2.0 Hz, 1 H, Ar in *E-isomer*), 8.52 (d, *J* = 1.6 Hz, 1H, Ar in *Z-isomer)*, 7.57 (d, *J* = 7.8 Hz, 1 H, Ar in *Z-isomer*), 7.51 (d, *J* = 8.1 Hz, 1 H, Ar in *E-isomer*), 7.42-7.33 (m, 3 H, Ar), 7.28-7.22(m, 2 H, Ar), 6.23 (q, *J* = 8.1 Hz, 1 H, C=C*H*CF_3 in *Z-isomer*) 6.16 (q, *J* = 8.0 Hz, 1 H, C=C*H*CF_3 in *E-isomer*). ^{13}**C NMR** (100 MHz, $CDCl_3$, rt): δ 150.5, 149.9, 149.6, 149.1 (q, *J* = 5.3 Hz), 148.8, 139.3, 136.6, 136.3, 136.0, 135.4, 133.3, 130.0, 129.1, 128.9, 128.5, 128.0, 123.4, 123.1, 122.9 (q, *J* = 269 Hz, *Z*-isomer), 122.8 (q, *J* = 270 Hz, *E*-isomer), 117.3 (q, *J* = 33.4 Hz, *Z*-isomer), 116.9 (q, *J* = 33.3 Hz, *E*-isomer). ^{19}**F NMR**

(376.5 MHz, $CDCl_3$, rt): δ -55.58 (d, *J* = 8.28 Hz, 3 F, *Z-isomer*), -55.99 (d, *J* = 8.28 Hz, 3 F, *E-isomer*). **HRMS** (ESI-TOF): calculated for $[C_{14}H_{10}F_3N+Na]^+$ requires 272.0658, found 272.0657.

1,1,1-Trifluoro-3,5-diphenyl-5-methyl-2-hexene (3i)

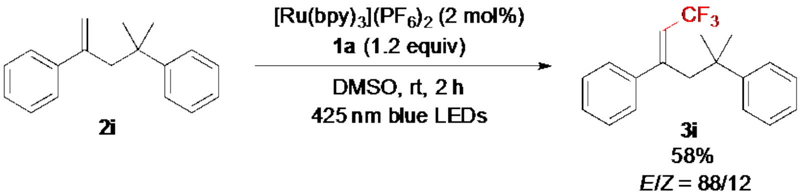

According to the general procedure, the title compound was synthesized from 2,4-diphenyl-4-methyl-1-pentene (**2i**, 59 mg, 0.25 mmol). The product was purified by silica gel flash column chromatography (hexane) to afford **3i** (45 mg, 58% yield, *E*/*Z* = 88:12). The stereochemistry was confirmed by ^{1}H NOESY NMR.

^{1}**H NMR** (400 MHz, $CDCl_3$, rt): δ 7.21-7.10 (m, 9 H, Ar), 7.06 (t, *J* = 7.0 Hz, Ar), 5.66 (q, *J* = 8.8 Hz, 1 H, C=C*H*CF_3 in *E*-isomer), 5.37 (q, *J* = 8.4 Hz, 1 H, C=C*H*CF_3 in *Z*-isomer), 3.09 (d, *J* = 1.2 Hz, 2 H, CCH_2C in *E*-isomer), 2.80 (d, *J* = 1.2 Hz, 2 H, CCH_2C in *Z*-isomer), 1.24 (s, 6 H, CH_3 in *Z*-isomer), 1.21 (s, 6 H, CH_3 in *E*-isomer). ^{13}**C NMR** (100 MHz, $CDCl_3$, rt): δ 152.7 (q, *J* = 5.4 Hz), 149.3, 141.8, 128.3, 128.0, 127.0, 125.8, 125.7, 123.4 (q, *J* = 269 Hz), 119.6 (q, *J* = 33.1 Hz), 44.5, 38.4, 28.8. ^{19}**F NMR** (376.5 MHz, $CDCl_3$, rt): δ –55.87 (d, *J* = 8.28 Hz, 3 F, *Z*-isomer), -55.96 (d, *J* = 9.41 Hz, 3 F, *E*-isomer). **HRMS** (ESI-TOF): calculated for $[C_{19}H_{19}F_3+Na]^+$ requires 327.1331, found 327.1332.

3,3,3-Trifluoro-1-(4-methoxyphenyl)-2-methyl-1-phenylpropene (3k)

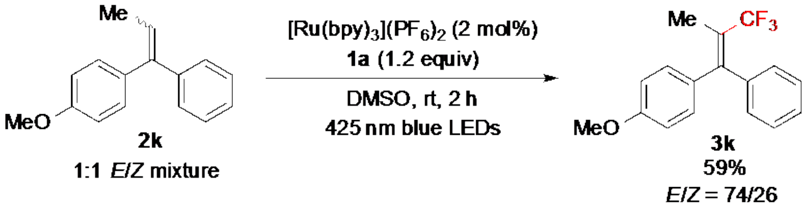

According to the general procedure, the title compound was synthesized from 1-methoxyphenyl-1-phenylpropene (**2k**). The product was purified by silica gel flash column chromatography (hexane→hexane/Et_2O = 29:1) to afford **3k** (45 mg, 59% yield, *E*/*Z* = 74/26). The stereochemistry was confirmed by ^{1}H NOESY NMR.

^{1}**H NMR** (400 MHz, $CDCl_3$, rt): δ 7.34-7.27 (m, 3 H, Ar), 7.14 (d, *J* = 9.6 Hz, 2 H, Ar in *E-isomer*), 7.12 (d, *J* = 8.4 Hz, 2 H, Ar in *Z-isomer*), 7.08 (d, *J* = 8.8 Hz, 2 H, Ar in *Z-isomer*), 7.05 (d, *J* = 8.8 Hz, 2 H, Ar in *E-isomer*), 6.85 (d, *J* = 8.8 Hz, 2 H, Ar in *E-isomer*), 6.82 (d, *J* = 8.8 Hz, 2 H, Ar in *Z-isomer*), 3.80 (s, 3 H, CH_3O in *E-isomer*), 3.79 (s, 3 H, CH_3O in *Z-isomer*),

1.96 (s, 3 H, CH_3CCF$_3$ in *E-isomer*), 1.90 (s, 3 H, CH_3CCF$_3$ in *Z-isomer*). ^{13}**C NMR** (100 MHz, CDCl$_3$, rt): δ 159.3, 159.3, 147.3, 147.3, 141.8, 141.4, 133.7, 133.4. 130.5, 130.0, 129.0, 128.7, 128.4, 128.0, 127.8, 127.6, 124.8 (q, J = 274 Hz), 123.0 (q, J = 27.9 Hz), 113.8, 113.5, 55.4, 55.3, 16.5. ^{19}**F NMR** (376.5 MHz, CDCl$_3$, rt): δ -59.23 (s, 3 F, *E-isomer*), -59.36 (s, 3 F, *Z-isomer*). **HRMS** (ESI-TOF): calculated for $[C_{17}H_{15}F_3O+Na]^+$ requires 315.0967, found 315.0963.

Procedures for the photocatalytic double C–H trifluoromethylation of alkenes

2-Trifluoromethyl-3,3,3-trifluoro-1-bis(4-methoxyphenyl)propene (4b)

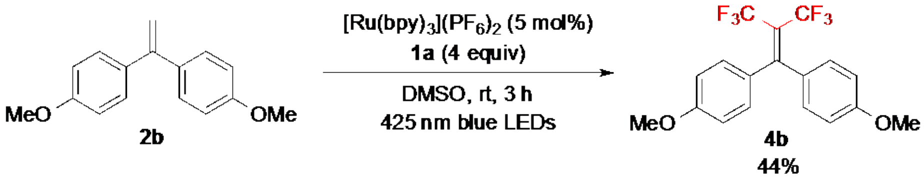

A 20 mL-Schlenk tube was charged with Umemoto's reagent (**1a**, 340 mg, 1.0 mmol, 4 equiv), $[Ru(bpy)_3](PF_6)_2$ (10.7 mg, 5 mol %), **2b** (60 mg, 0.25 mmol), and DMSO (5 mL) under N_2. The tube was irradiated for 3 h at room temperature (water bath) with stirring by 3 W blue LED lamps ($h\nu$ = 425 nm ± 15 nm) placed at a distance of 2–3 cm. After the reaction, H_2O was added. The resulting mixture was extracted with Et_2O, washed with H_2O, dried (Na_2SO_4), and filtered. The filtrate was concentrated in vacuo and the residue was purified by flash column chromatography on silica gel (hexane→hexane/Et_2O = 29:1) to afford **4b** as a product mixture with **3b**. Further purification by GPC provided pure **4b** in 44% isolated yield.

^{1}**H NMR** (400 MHz, CDCl$_3$, rt): δ 7.09 (d, J = 8.8 Hz, 4 H, Ar), 6.87 (d, J = 8.8 Hz, 4 H, Ar), 3.83 (s, 6 H, CH_3O). ^{13}**C NMR** (100 MHz, CDCl$_3$, rt): δ 161.3, 160.1, 132.3, 131.2, 122.3, (q, J = 273 Hz), 115.7 (q, J = 30.1 Hz), 113.7, 55.4. ^{19}**F NMR** (376.5 MHz, CDCl$_3$, rt): δ -54.80 (s, 6 F). **HRMS** (ESI-TOF): calculated for $[C_{18}H_{14}F_6O_2+Na]^+$ requires 399.0790, found 399.0795.

1-Bis(4-bromophenyl)-2-trifluoromethyl-3,3,3-trifluoropropene (4d)

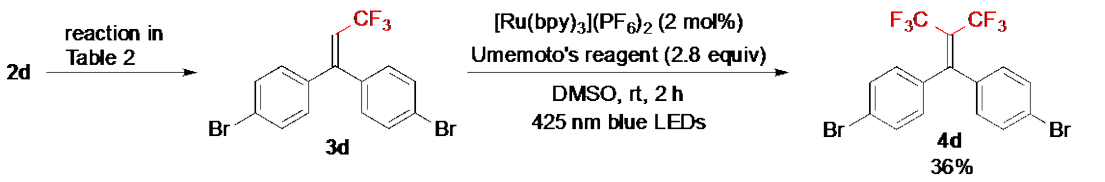

Two-step reactions were conducted. According to the procedure in Table 2, **2d** (85 mg, 0.25 mmol) was converted into the mixture of **3d** and a small amount of **4d** (90 mg). To the mixture, Umemoto's reagent (**1a**, 238 mg), $[Ru(bpy)_3](PF_6)_2$ (4.3 mg) and DMSO (5 mL) were added under N_2. The solution was placed at a distance of 2–3 cm from the 3 W blue LED lamp ($h\nu$ =

425 nm ± 15 nm) and stirred at room temperature (water bath) for 2 h. After the reaction, H_2O was added. The resulting mixture was extracted with Et_2O, washed with H_2O, dried (Na_2SO_4), and filtered. The filtrate was concentrated in vacuo. The residue was treated by mCPBA (0.17 g, ca. 0.7 mmol) in CH_2Cl_2 to convert the dibenzothiophene to sulfoxide, which was more easily separated from the products. After the solution was stirred at room temperature for 2 h, an aqueous solution of $Na_2S_2O_3 \cdot 5H_2O$ was added to the solution, which was extracted with CH_2Cl_2. The organic layer was washed with H_2O, dried (Na_2SO_4), and filtered. The filtrate was concentrated in vacuo and the residue was purified by chromatography on silica gel (eluent: hexane) to afford **4d** as a product mixture with **3d**. Further purification by GPC provided pure **4d** in 36% isolated yield.

^{1}H NMR (400 MHz, $CDCl_3$, rt): δ 7.51 (d, J = 8.4 Hz, 4 H, Ar), 7.01 (d, J = 8.4 Hz, 4 H, Ar).
^{13}C NMR (100 MHz, $CDCl_3$, rt): δ 157.9, 137.7, 131.9, 130.3, 124.9, 121.5 (q, J = 276 Hz), 119.4 (q, J = 31.0 Hz), **^{19}F NMR** (376.5 MHz, $CDCl_3$, rt): δ -55.31 (s, 6 F). **HRMS** (ESI-TOF): calculated for $[C_{16}H_8Br_2F_6+Na]^+$ requires 474.8950, found 474.8953.

Time profile of the photocatalytic C−H trifluoromethylation of 2a

The trifluoromethylation of **2a** was performed with/without visible light irradiation. The time profile is shown in Figure S3. As a result, continuous irradiation of visible light is essential for efficient reaction. Furthermore, the result of this experiment suggests that radical chain propagation is not the main component in this reaction.

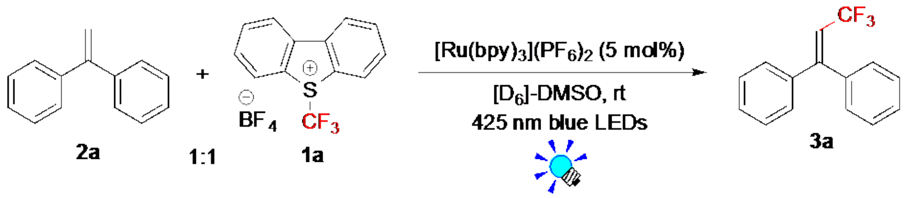

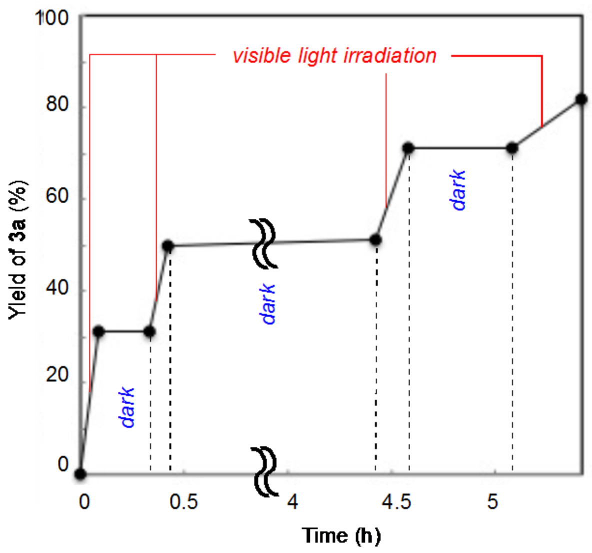

Figure S3: Time profile of the photocatalytic C−H trifluoromethylation of **2a**

^{1}H, ^{13}C NMR spectra

Assignment of stereochemistry of product 3

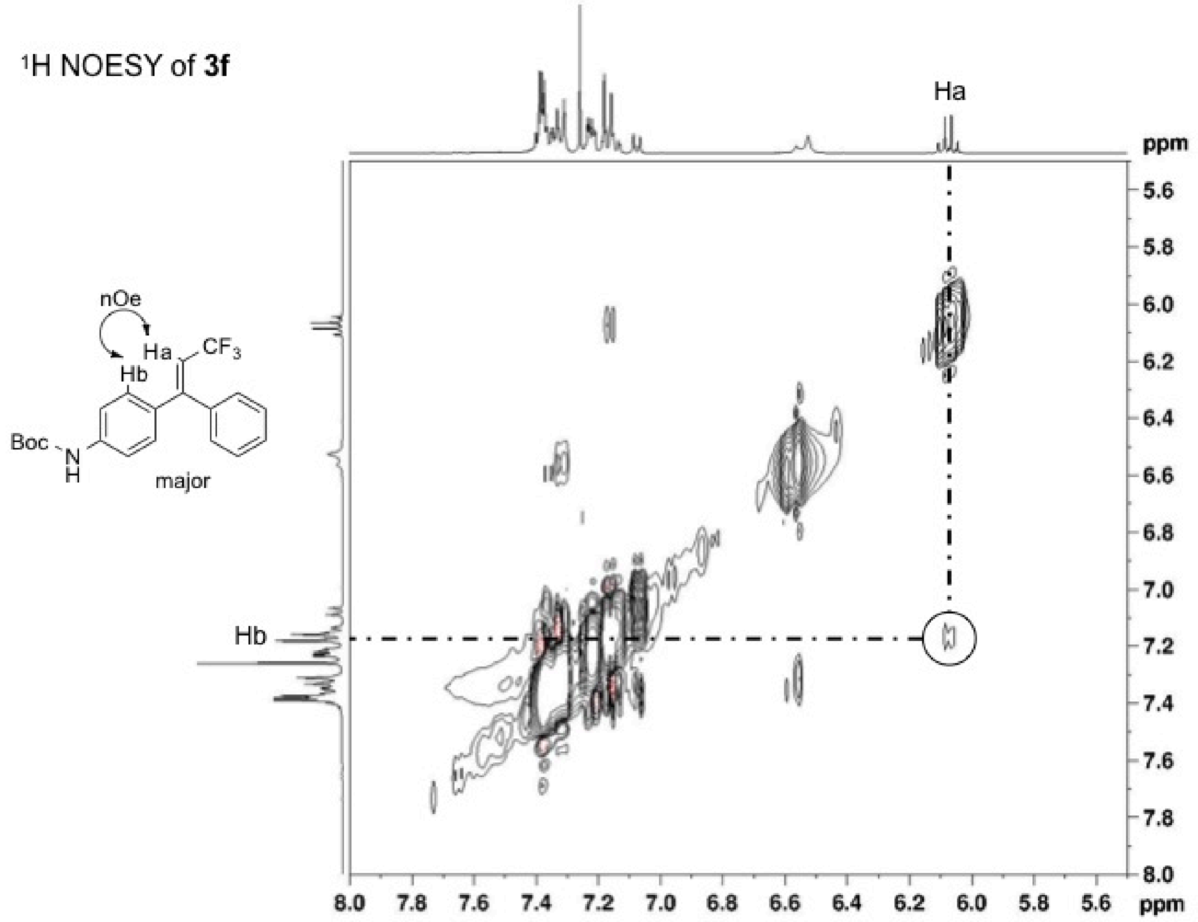

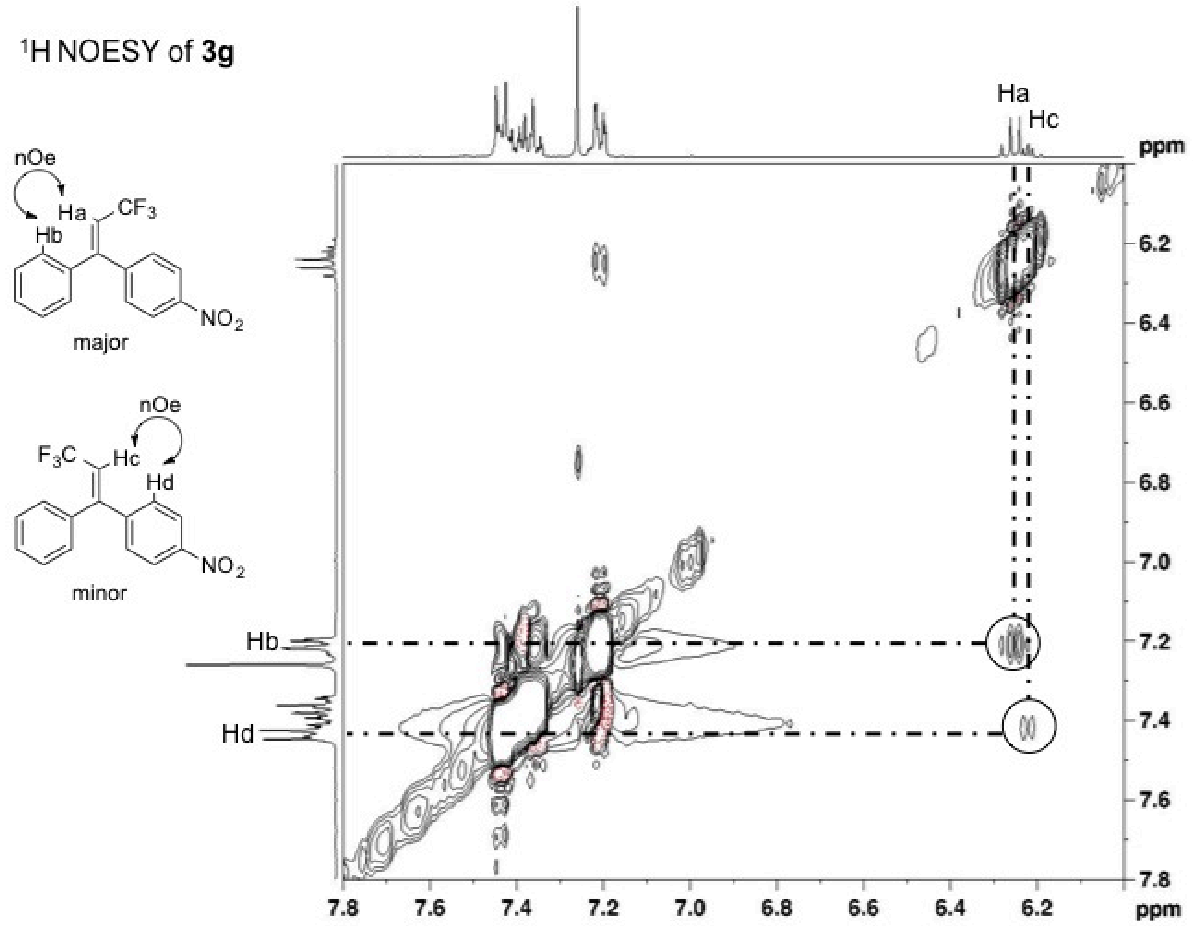

^{1}H NOESY of **3g**
nOe
Ha
CF3
Hb
NO2
major
nOe
F3C
Hc
Hd
NO2
minor
Ha
Hc
ppm
Hb
Hd
6.2
6.4
6.6
6.8
7.0
7.2
7.4
7.6
7.8
7.8
7.6
7.4
7.2
7.0
6.8
6.6
6.4
6.2
ppm

^{1}H NOESY **3h**

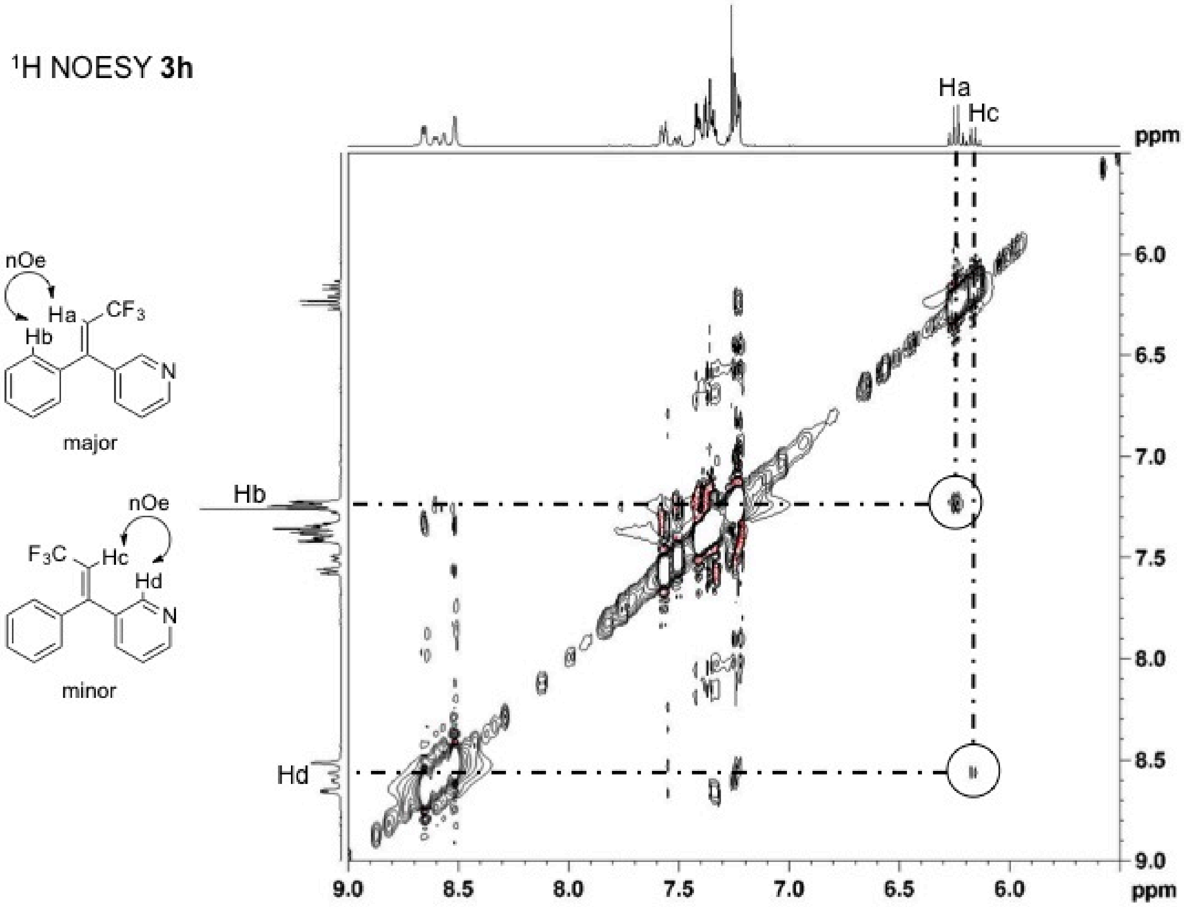
nOe
Ha
CF3
Hb
N
major
nOe
F3C
Hc
Hd
N
minor
Hb
Hd
Ha
Hc
ppm
6.0
6.5
7.0
7.5
8.0
8.5
9.0
ppm
9.0
8.5
8.0
7.5
7.0
6.5
6.0
ppm

^{1}H NOESY of **3i**

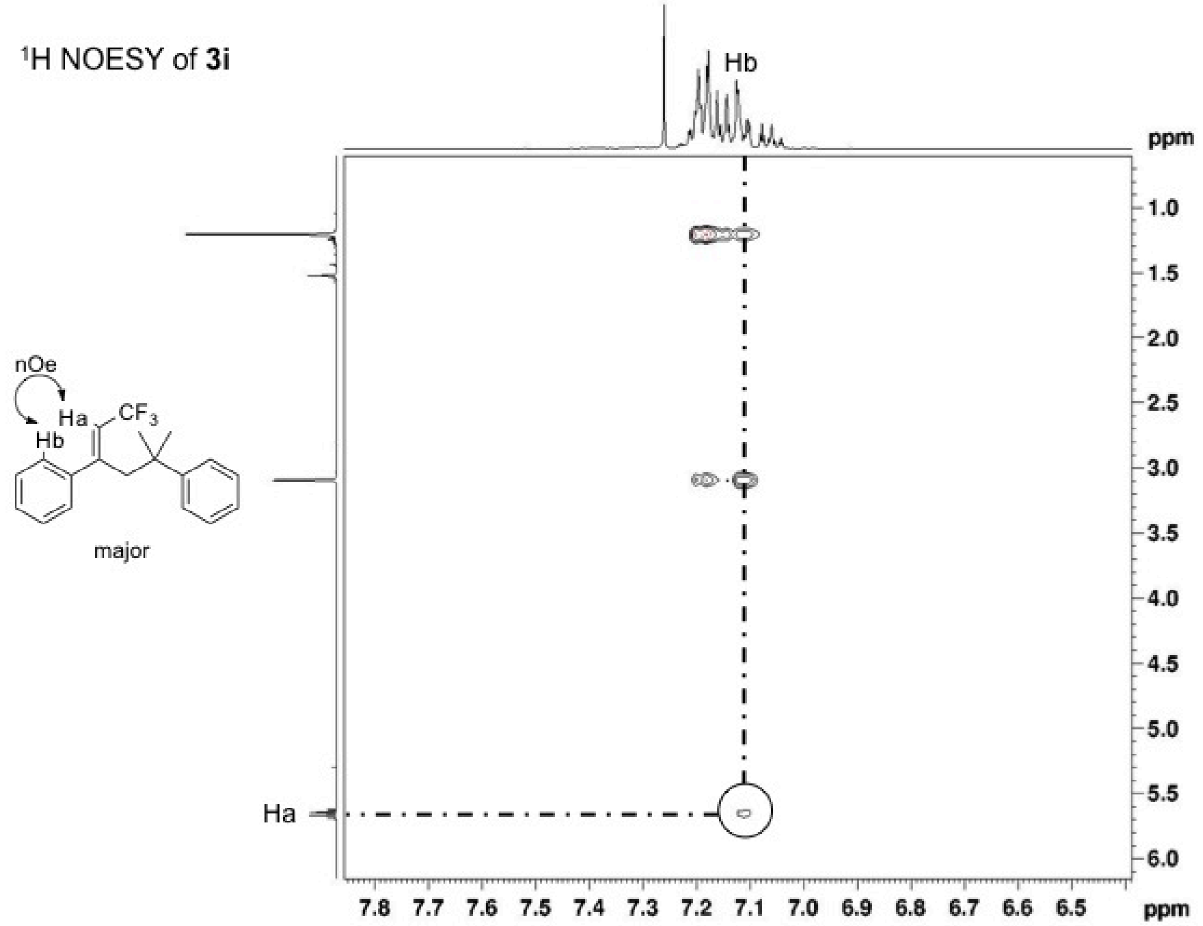

^{1}H NOESY of **3k**

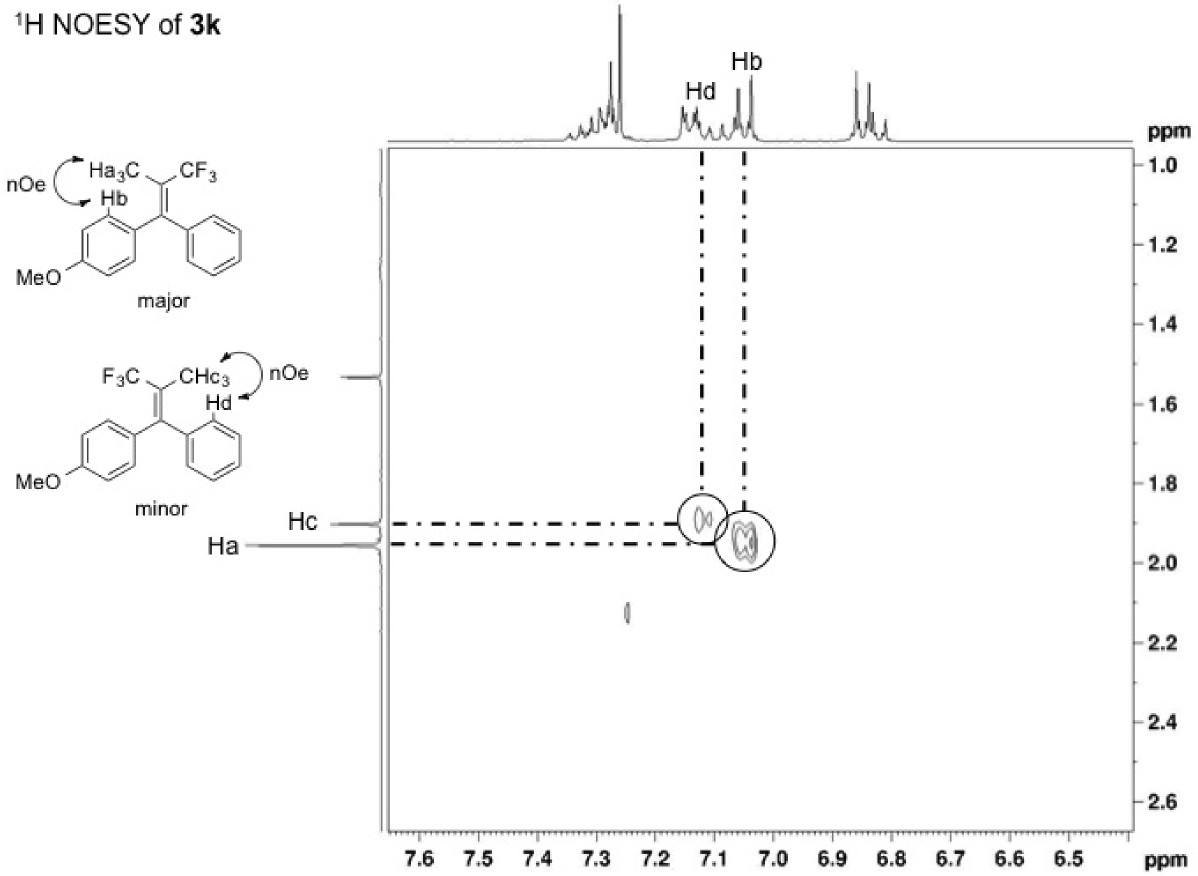

References

[1] Ross, H. B.; Boldaji, M.; Rillema, D. P.; Blanton, C. B.; White, R. P. *Inorg. Chem.* **1989**, *28*, 1013.

[2] Tamayo, A. B.; Alleyne, B. D.; Djurovich, P. I.; Lamansky, S.; Tsyba, I.; Ho, N. N.; Bau, R.; Thompson, M. E. *J. Am. Chem. Soc*. **2003**, *125*, 7377.

[3] Nunes, C. M.; Limberger, J.; Poersh, S.; Seferin, M.; Monteiro, A. L. *Synthesis* **2009**, *16*, 2761.

[4] Cho, E. J.; Buchwald, S. L. *Org. Lett*. **2011**, *13*, 6552.

[5] Alkhafaji, H. M. H.; Ryabukhin, D. S.; Muzalevskiy, V. M.; Vasilyev, A. V.; Fukin, G. K.; Shastin, A. V.; Nenajdenko, V. G. *Eur. J. Org. Chem*. **2013**, 1132.

[6] Fuchibe, K.; Atobe, K.; Fujita, Y.; Mori, K.; Akiyama, T. *Chem. Lett*. **2010**, *39*, 867.

[7] Liu, X.; Shimizu, M.; Hiyama, T. *Angew. Chem. Int. Ed*. **2004**, *43*, 879.

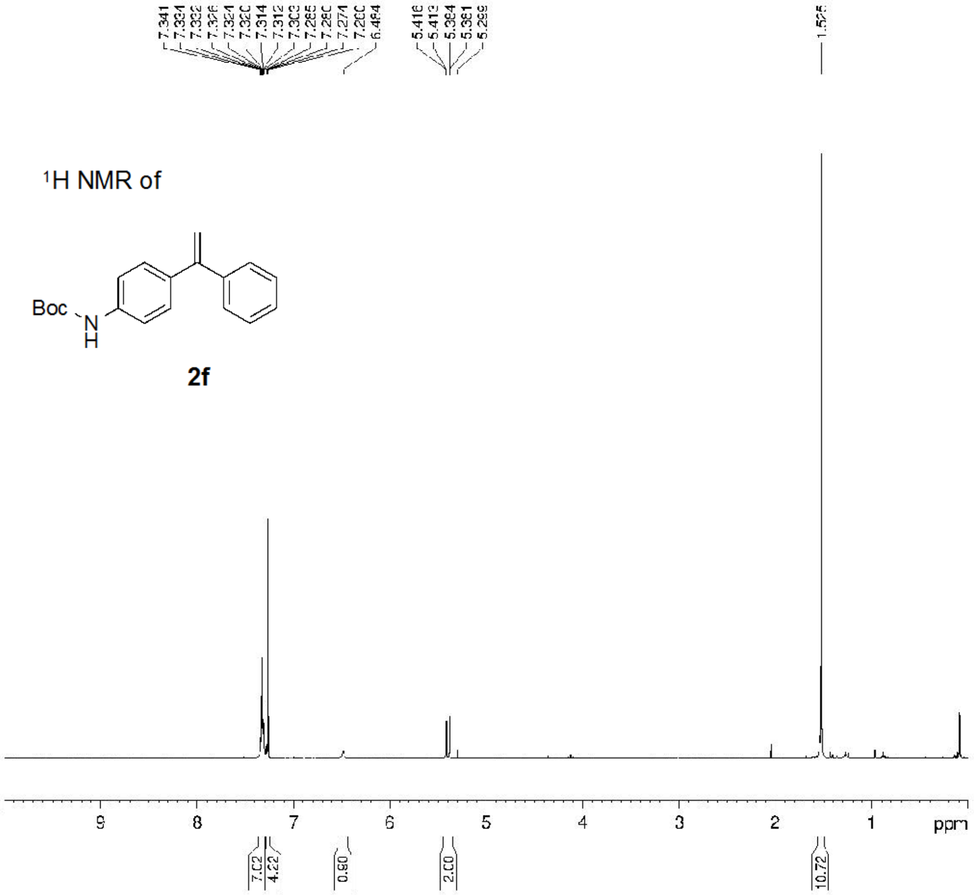
1H NMR of
Boc
N
H
2f
ppm

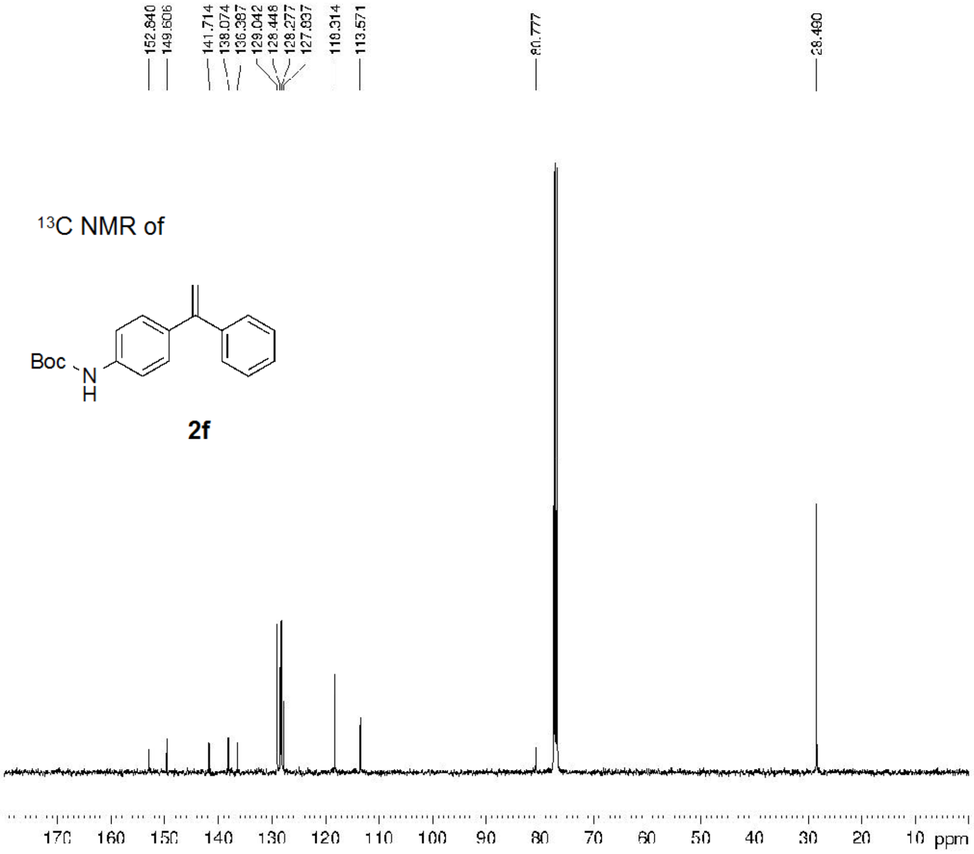
13C NMR of
Boc
N
H
2f
152.840
149.606
141.714
138.074
136.367
129.042
128.448
128.277
127.837
118.314
113.571
80.777
28.490
ppm

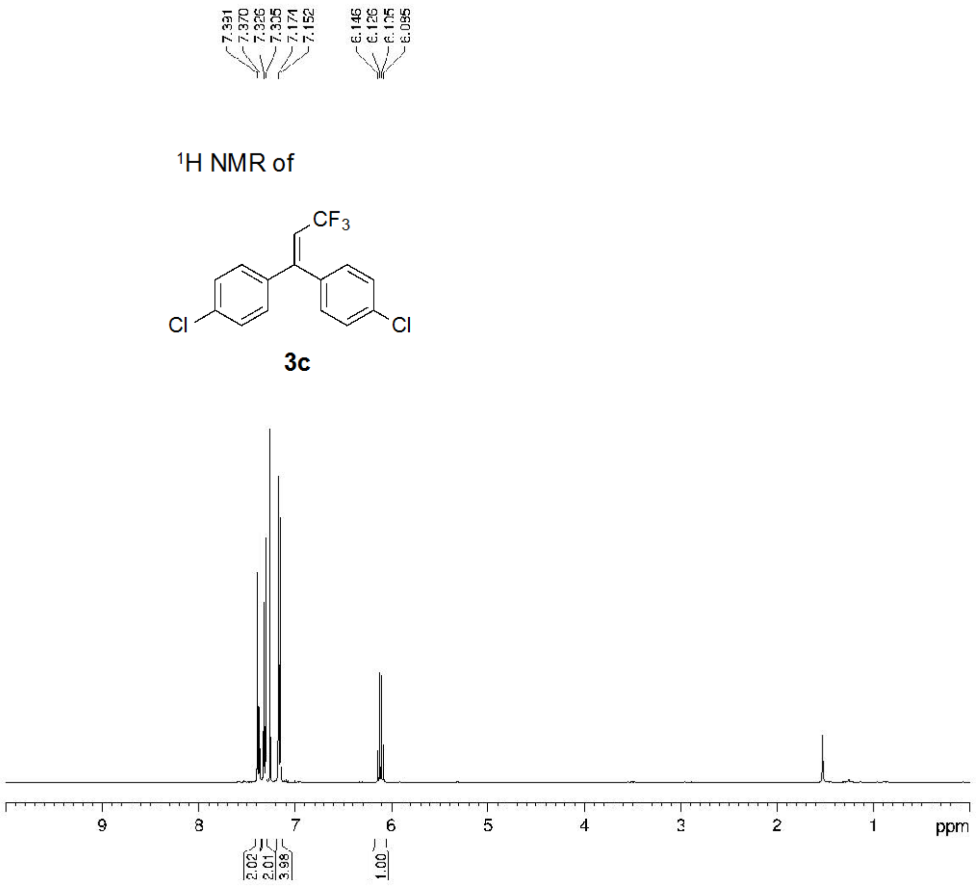
7.391
7.370
7.326
7.305
7.174
7.152
6.146
6.126
6.105
6.085
1H NMR of
CF3
Cl
Cl
3c
9 8 7 6 5 4 3 2 1 ppm
2.02
2.01
3.98
1.00

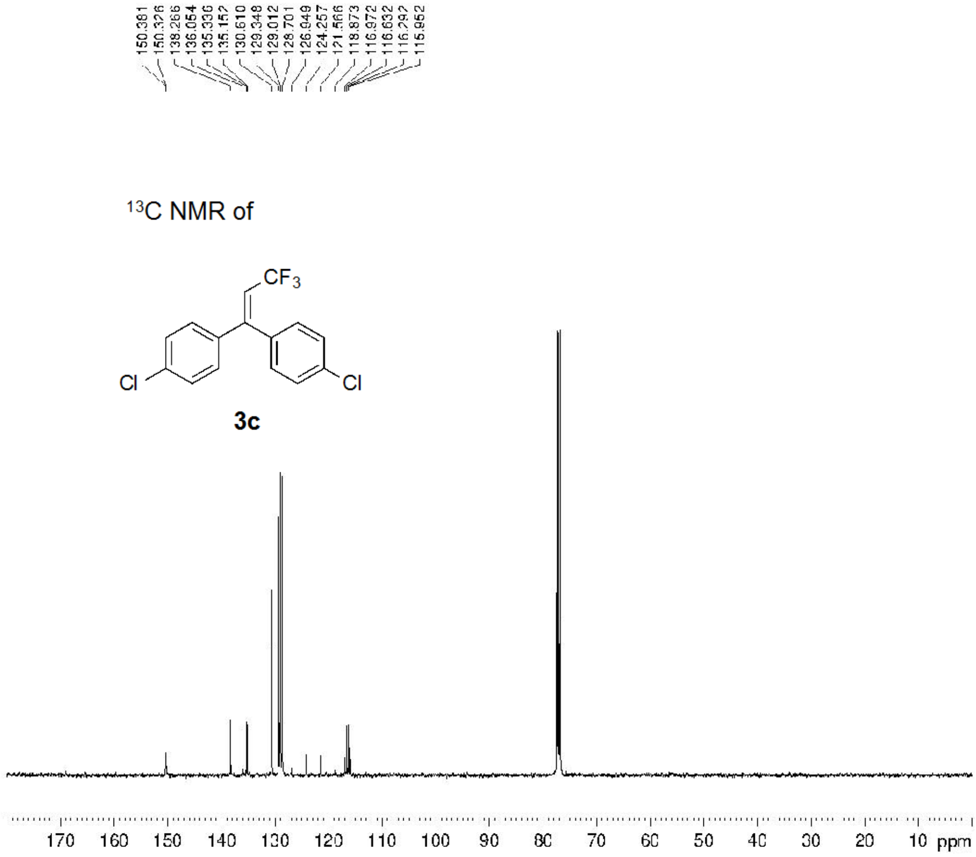
150.381
150.326
138.266
136.054
135.336
135.152
130.610
129.348
129.012
128.701
126.949
124.257
121.566
118.873
116.972
116.632
116.292
115.952
13C NMR of
CF3
Cl
Cl
3c
170 160 150 140 130 120 110 100 90 80 70 60 50 40 30 20 10 ppm

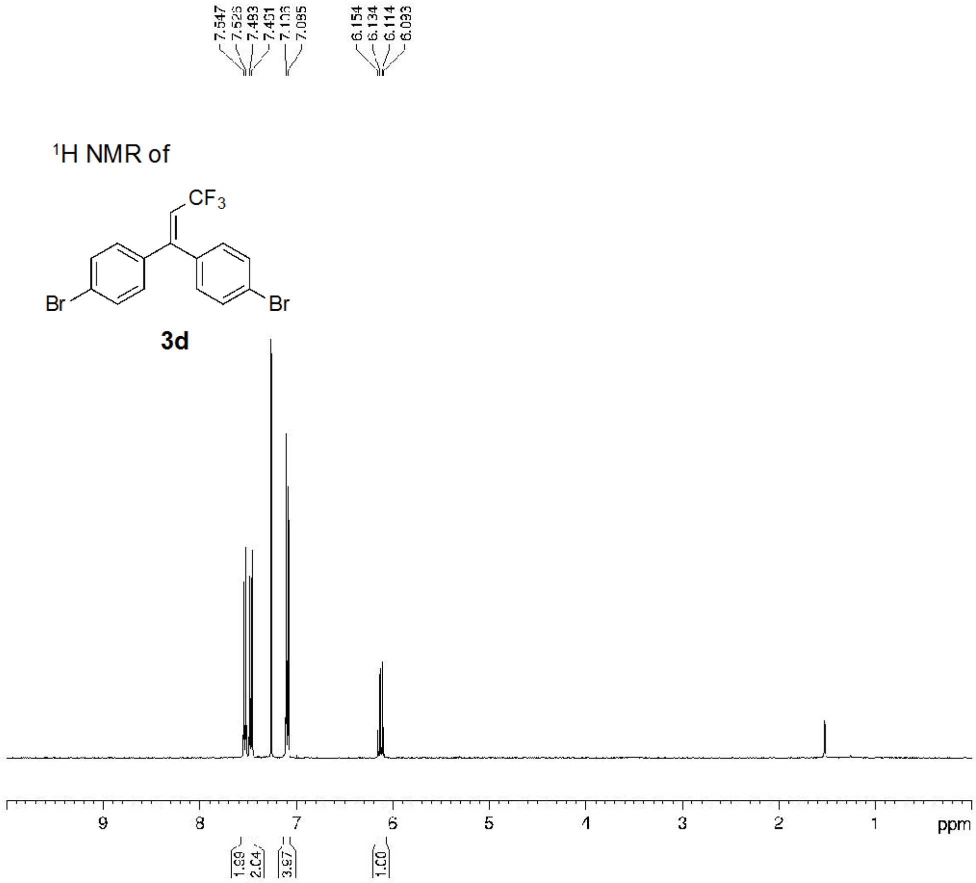
7.547
7.526
7.493
7.471
7.106
7.085
6.154
6.134
6.114
6.093
1H NMR of
CF3
Br
Br
3d
9
8
7
6
5
4
3
2
1
ppm
1.99
2.04
3.97
1.00

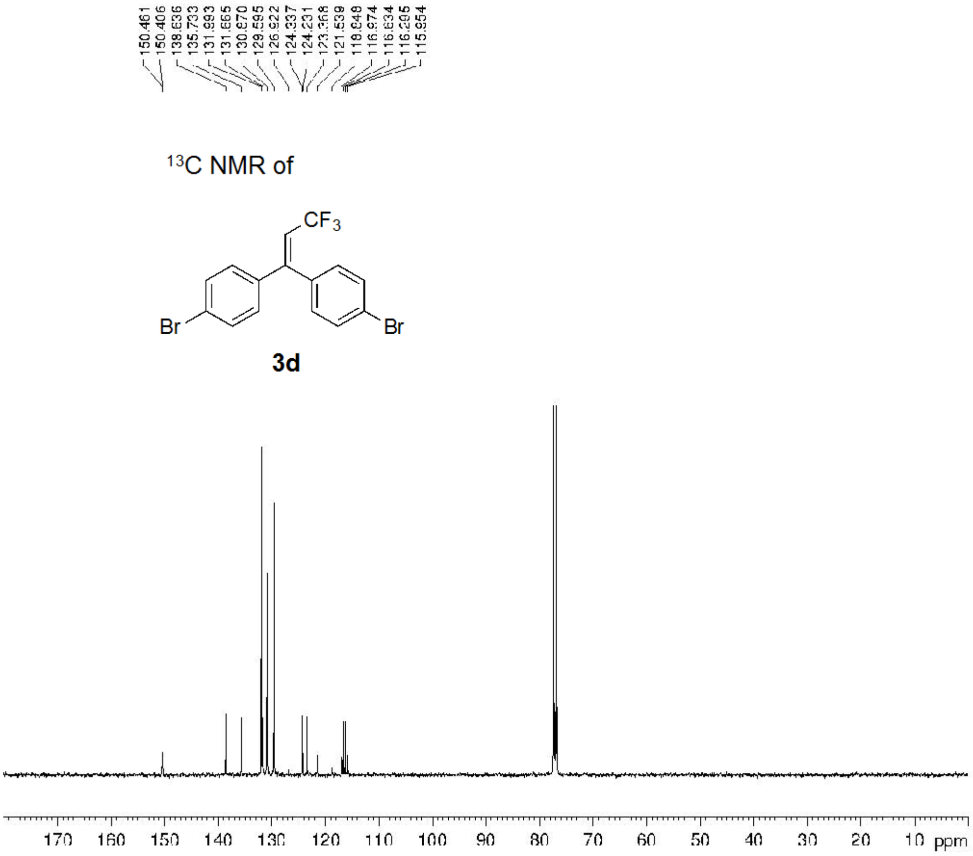
150.461
150.406
138.636
135.733
131.893
131.865
130.870
129.595
126.922
124.337
124.231
123.368
121.539
118.848
116.874
116.634
116.295
115.954
13C NMR of
CF3
Br
Br
3d
170
160
150
140
130
120
110
100
90
80
70
60
50
40
30
20
10
ppm

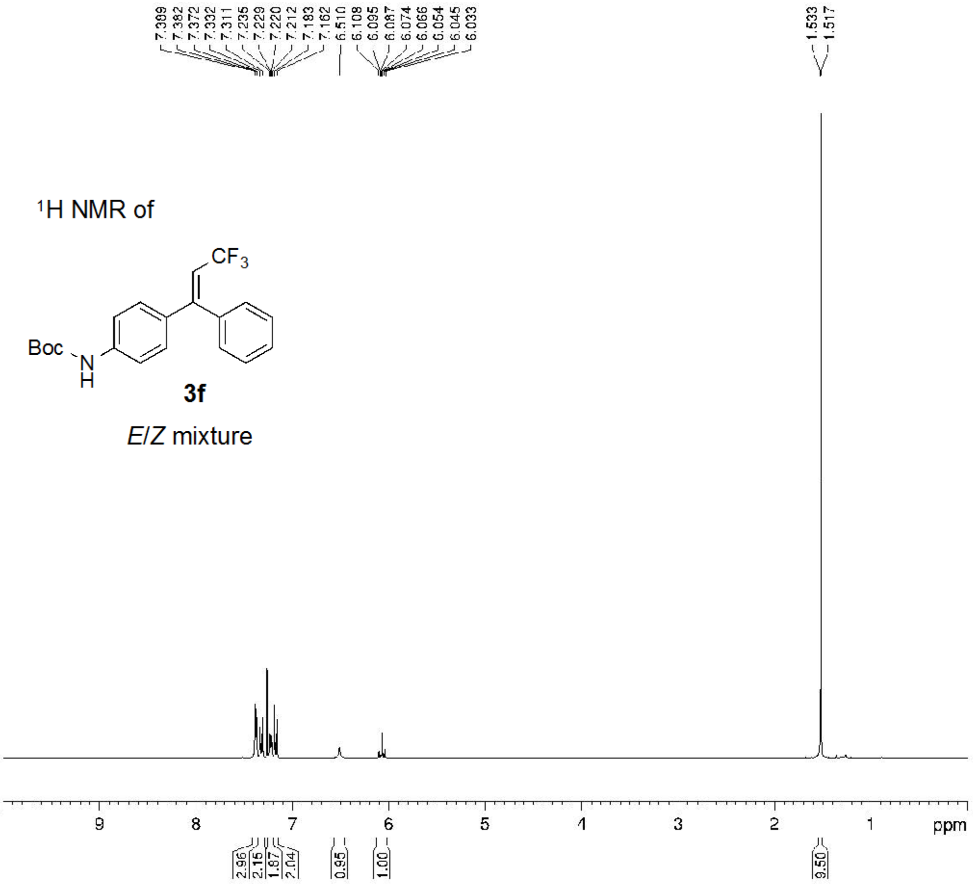
7.389
7.382
7.372
7.332
7.311
7.235
7.229
7.220
7.212
7.183
7.162
6.510
6.108
6.095
6.087
6.074
6.066
6.054
6.045
6.033
1.533
1.517
1H NMR of
CF3
Boc
N
H
3f
E/Z mixture
9
8
7
6
5
4
3
2
1
ppm
2.96
2.15
1.87
2.04
0.95
1.00
9.50

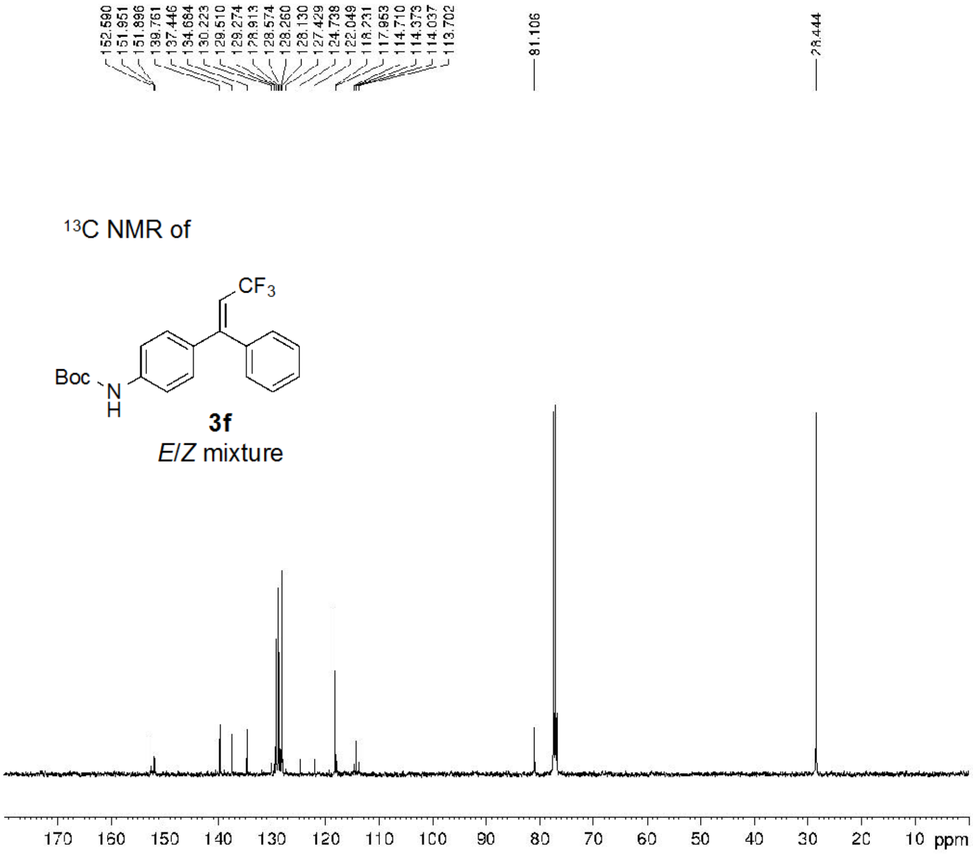
152.590
151.951
151.896
139.761
137.446
134.684
130.223
129.510
129.274
128.913
128.574
128.260
128.130
127.429
124.738
122.049
118.231
117.953
114.710
114.373
114.037
113.702
81.106
28.444
13C NMR of
CF3
Boc
N
H
3f
E/Z mixture
170
160
150
140
130
120
110
100
90
80
70
60
50
40
30
20
10
ppm

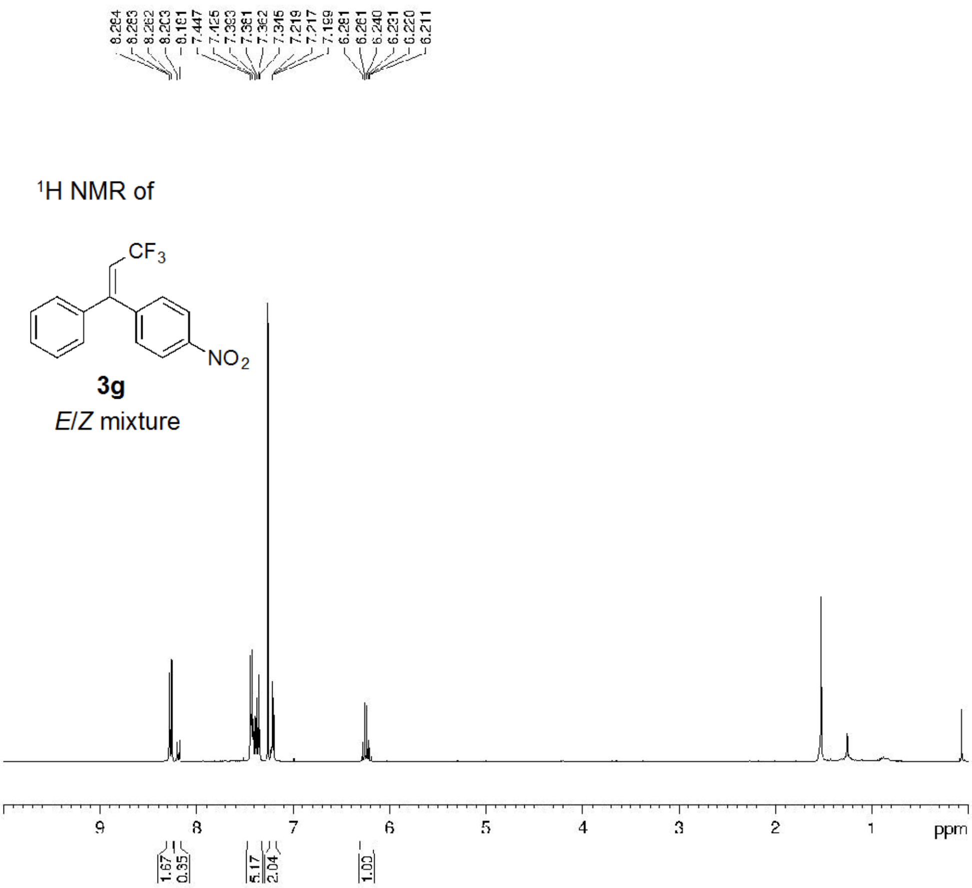
1H NMR of
CF3
NO2
3g
E/Z mixture
ppm

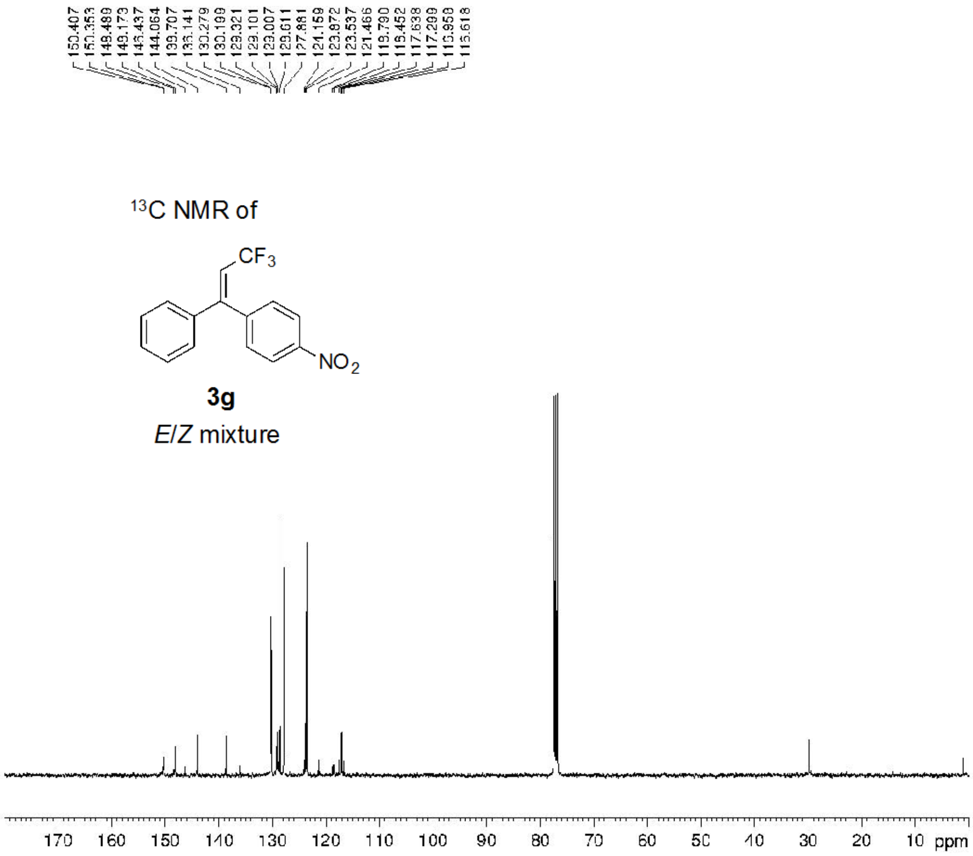
13C NMR of
CF3
NO2
3g
E/Z mixture
ppm

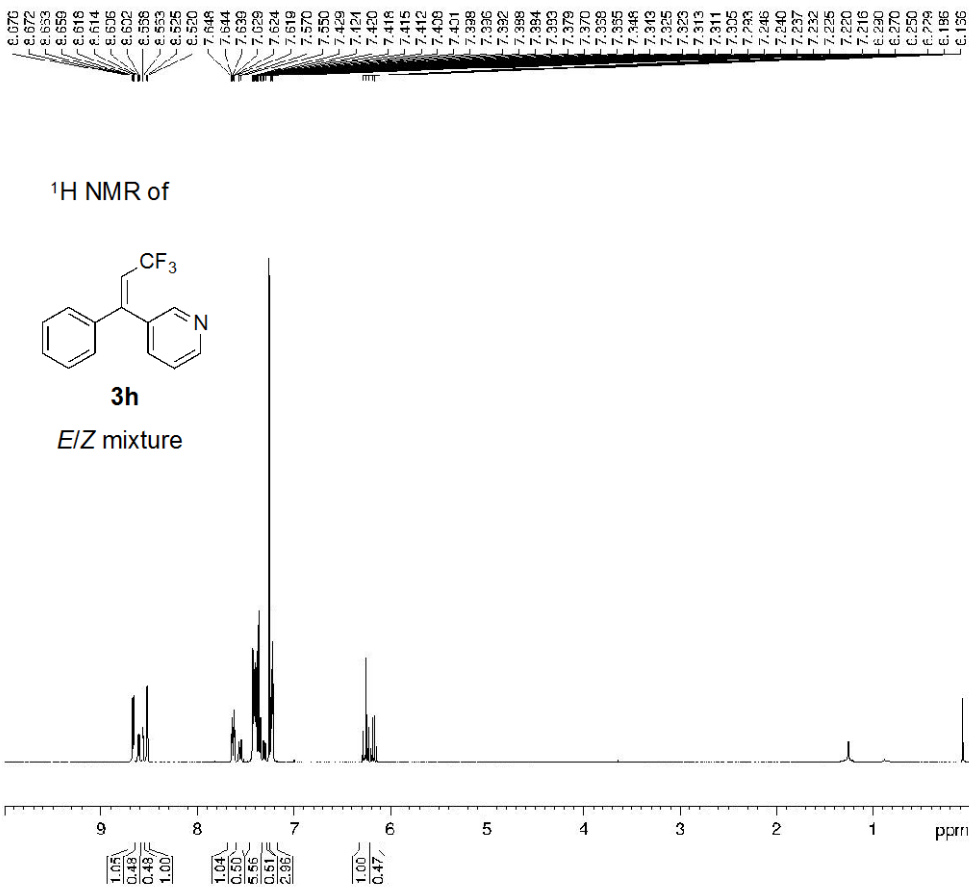

¹H NMR of
CF3
N
3h
E/Z mixture
ppm

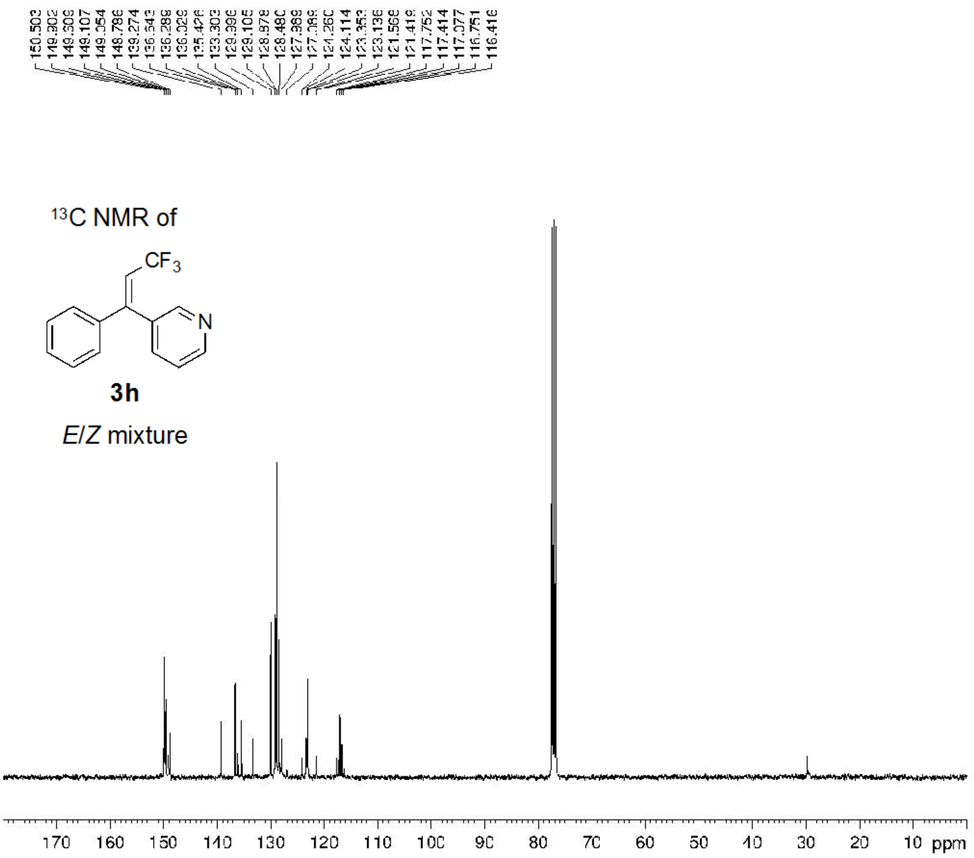

¹³C NMR of
CF3
N
3h
E/Z mixture
ppm

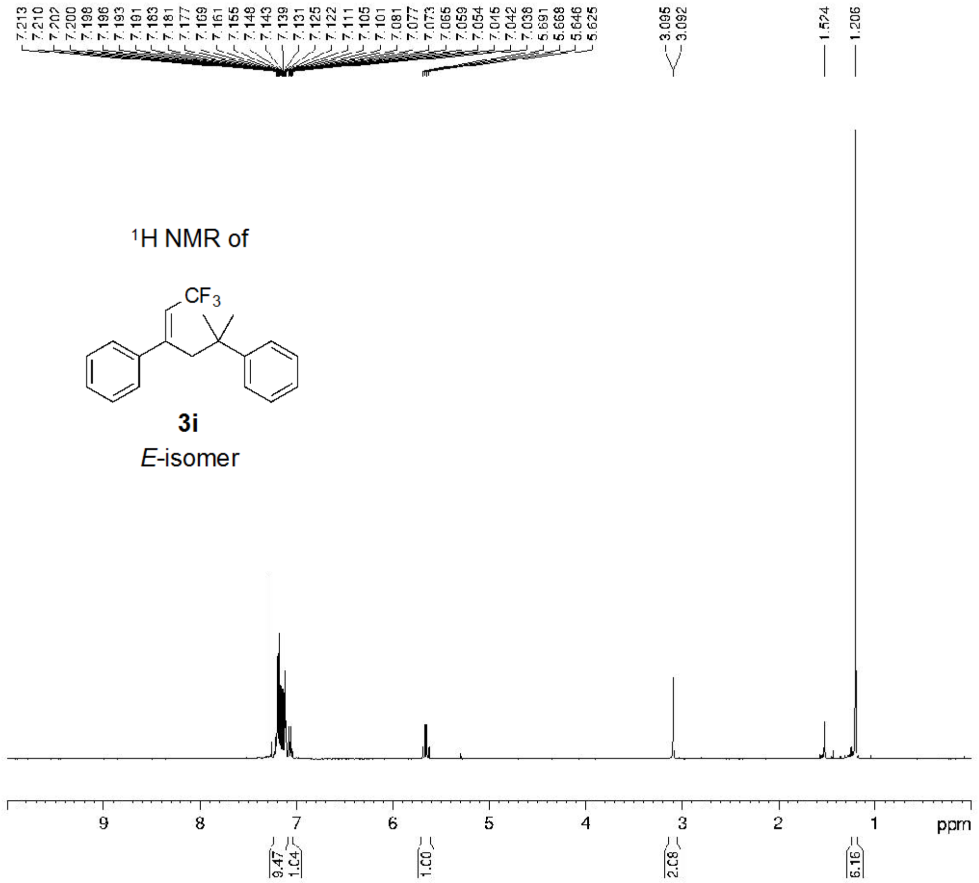
1H NMR of
CF3
3i
E-isomer
ppm

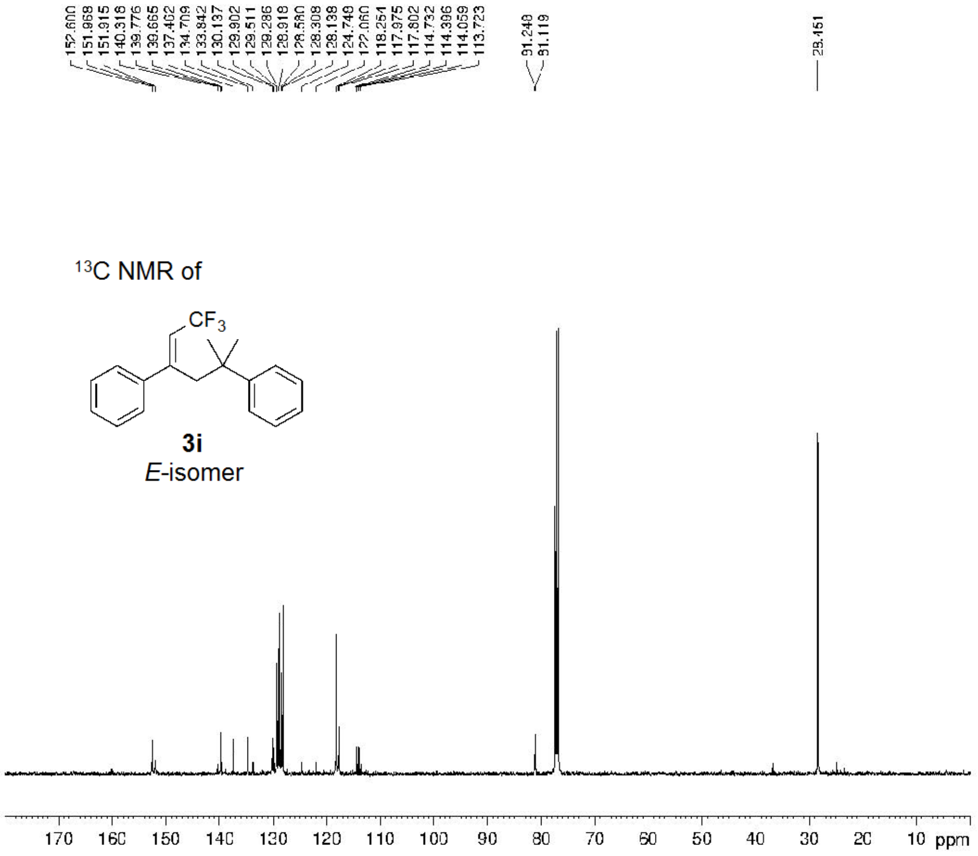
13C NMR of
CF3
3i
E-isomer
ppm

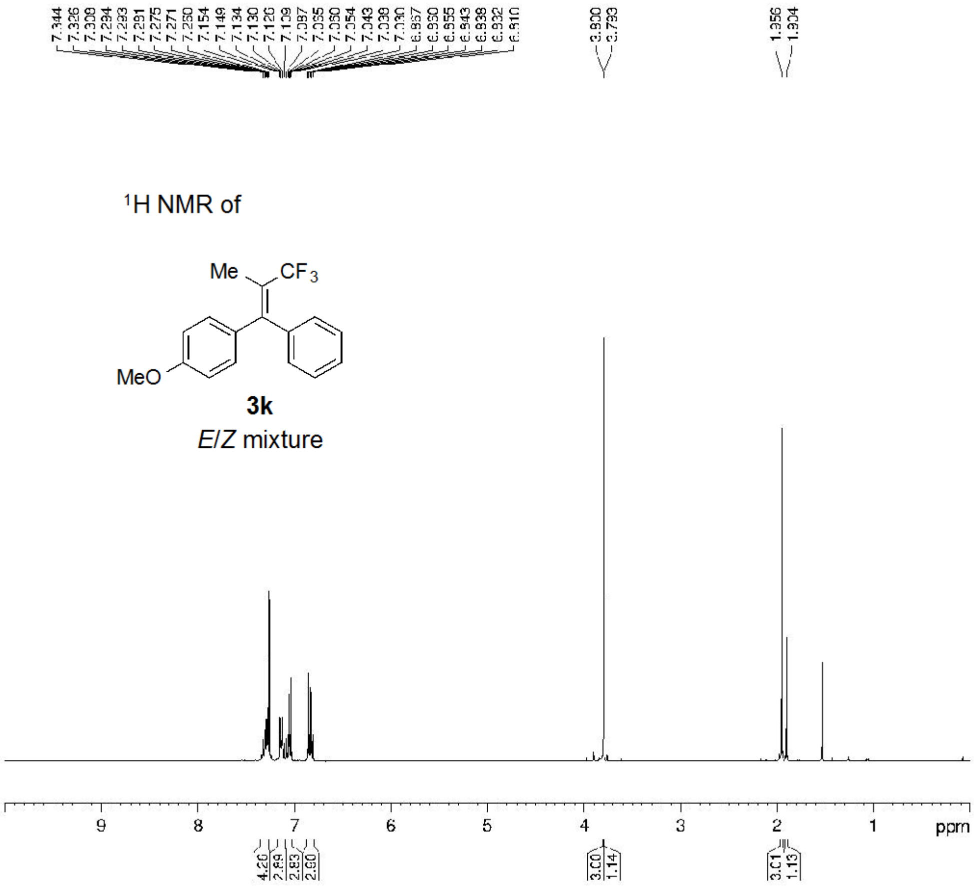
7.344 7.326 7.309 7.294 7.293 7.291 7.275 7.271 7.260 7.154 7.149 7.134 7.130 7.126 7.109 7.087 7.065 7.060 7.054 7.043 7.039 7.030 6.957 6.950 6.955 6.943 6.938 6.932 6.910
3.800 3.793
1.956 1.934
^{1}H NMR of
Me
CF_3
MeO
3k
E/Z mixture
9 8 7 6 5 4 3 2 1 ppm
4.26 2.89 2.83 2.90
3.00 1.14
3.01 1.13

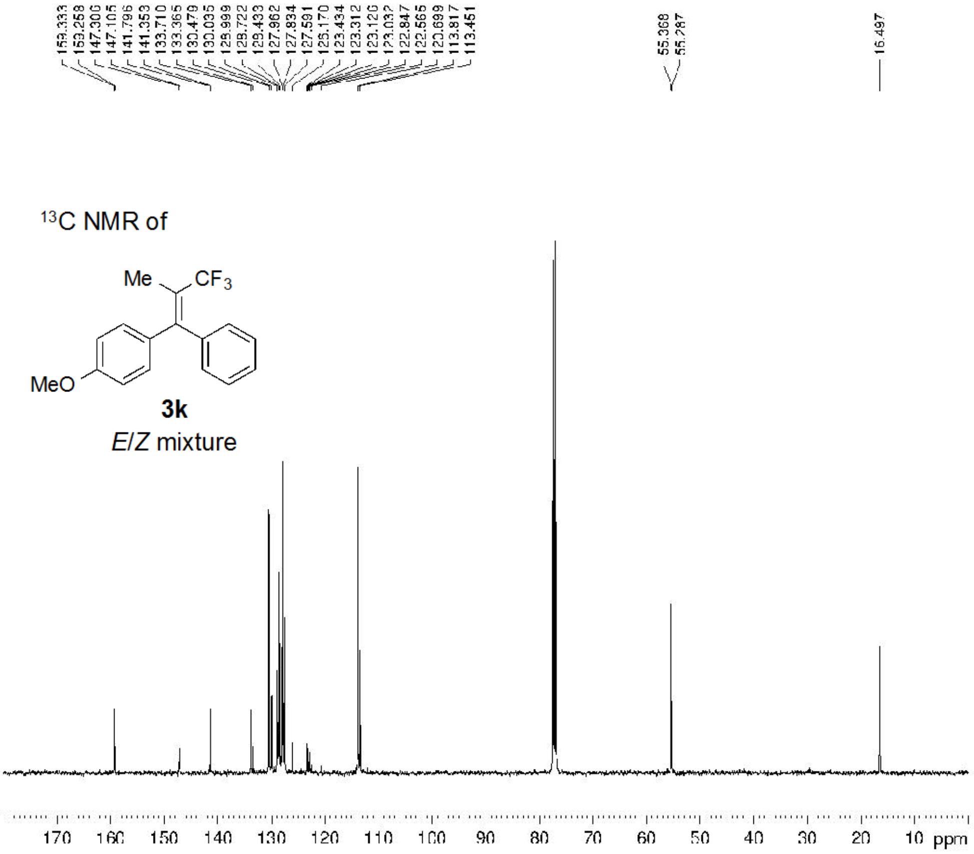
159.333 159.258 147.306 147.105 141.796 141.353 133.710 133.365 130.479 130.035 129.999 128.722 128.433 127.962 127.834 127.591 125.170 123.434 123.312 123.126 123.032 122.847 122.565 120.699 113.817 113.451
55.368 55.287
16.497
^{13}C NMR of
Me
CF_3
MeO
3k
E/Z mixture
170 160 150 140 130 120 110 100 90 80 70 60 50 40 30 20 10 ppm

^{1}H NMR of

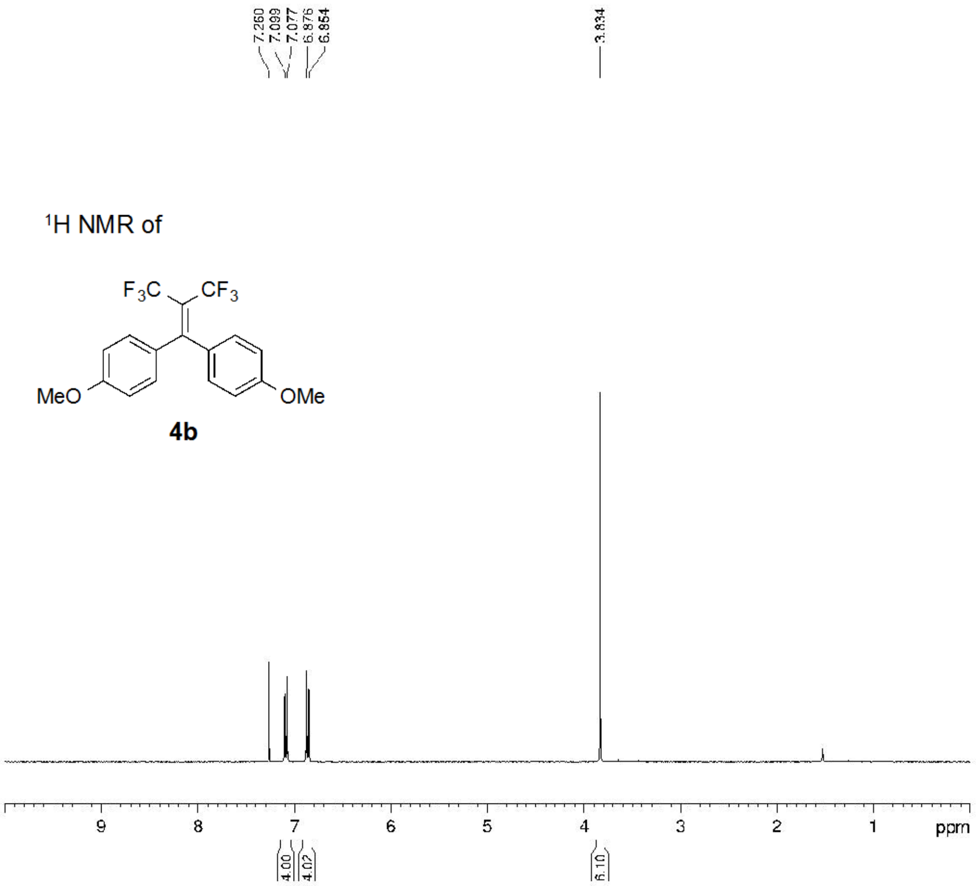

^{13}C NMR of

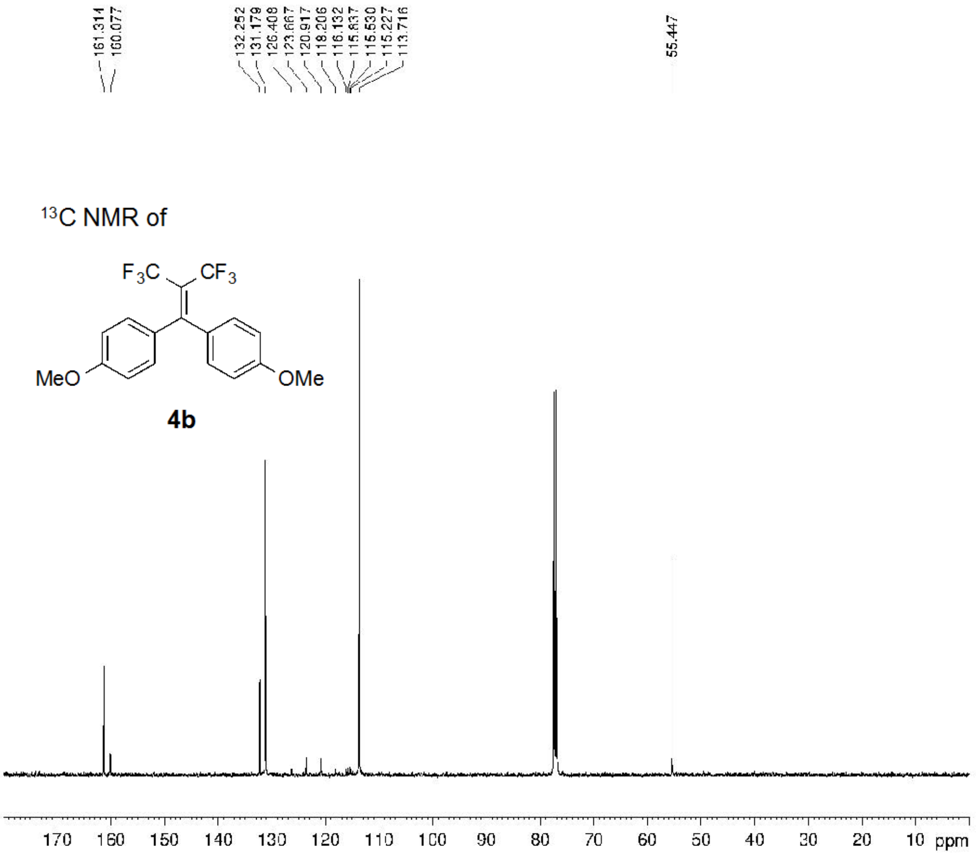

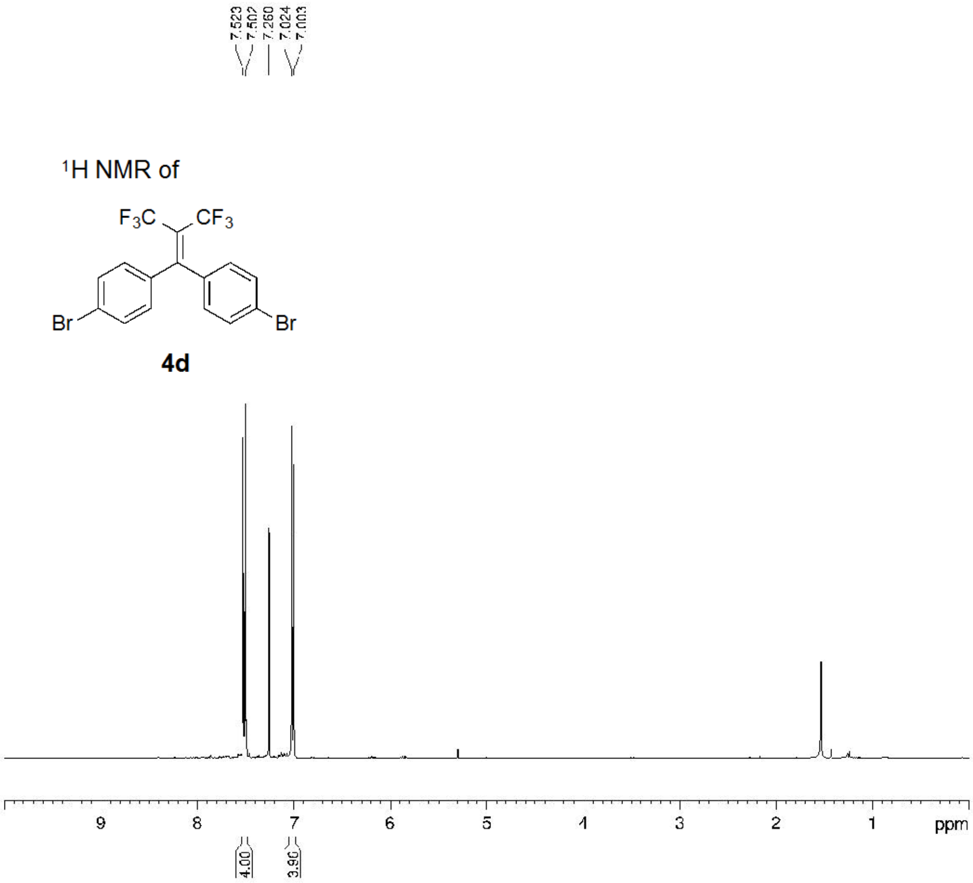
7.523
7.502
7.260
7.024
7.003
1H NMR of
F3C
CF3
Br
Br
4d
9
8
7
6
5
4
3
2
1
ppm
4.00
3.90

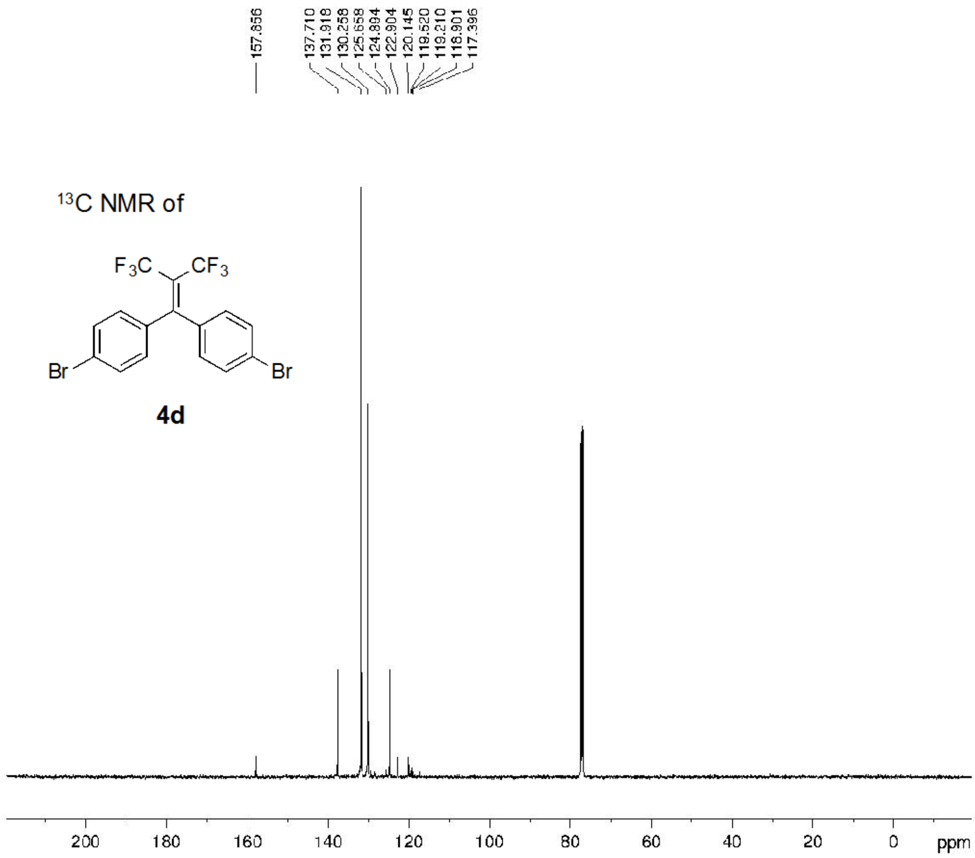
157.856
137.710
131.918
130.258
125.658
124.894
122.904
120.145
119.520
119.210
118.901
117.396
13C NMR of
F3C
CF3
Br
Br
4d
200
180
160
140
120
100
80
60
40
20
0
ppm
